# Facile Synthesis of High Areal Density and Stable Pt Single‐Atom Electrocatalysts by Arc Discharge Atomization and CoNi Trapping

**DOI:** 10.1002/advs.202511806

**Published:** 2025-08-27

**Authors:** Hongzhe He, Xiaoqiong Ren, Ruoqun Zhang, Sasha Yang, Jinxing Gu, Ke Wang, Binbin Qian, Ning Chen, Lian Zhang, Jianglong Yu, Yuan Cheng, Baiqian Dai

**Affiliations:** ^1^ Department of Chemical and Biological Engineering Monash University Victoria 3800 Australia; ^2^ Innovation Center for Chemical Sciences College of Chemistry Chemical Engineering and Materials Science Soochow University Suzhou Jiangsu 215123 China; ^3^ Department of Materials Science and Engineering Monash University Clayton VIC 3800 Australia; ^4^ Suzhou Industry Park Monash Suzhou Research Institute Suzhou Industry Park Suzhou 215123 China; ^5^ School of Automation Xi'an University of Posts & Telecommunications Shaanxi 71021 China; ^6^ School of Chemistry and Environmental Engineering Yancheng Teachers University Yancheng 224002 China

**Keywords:** carbon nanotubes, direct current arc discharge (DCAD), single‐atom catalysts (SACs), water splitting

## Abstract

Pt‐group metal single‐atom catalysts (SACs) with a large single‐atom areal density are highly desirable for efficient electrocatalysis but remain challenging to synthesize. Herein, a facile vacuum direct current arc discharge (DCAD) strategy is reported for the rapid and scalable synthesis of Pt SACs with an unprecedented areal density of 10.6 atoms nm^−2^ (3.82 wt.% Pt loading) firmly anchored on CoNi nanoalloy and confined by carbon nanotubes (CoNiPt_SA_@G). Notably, due to the strong electron trapping effect between Pt SA and CoNi substrates, CoNiPt_SA_@G retains its structural integrity at 1000 °C, demonstrating an outstanding thermal stability despite the ultra‐high areal density. Moreover, the DCAD strategy is universal, which can be applied to other metals such as Iridium. It is also scalable, with a demonstrated gram‐scale yield achieved within 0.5 h. The resulting CoNiPt_SA_@G catalyst exhibits exceptional hydrogen evolution reaction performance, achieving an overpotential of 23 mV at 10 mA cm^−2^, a mass activity over 5 times higher than that of 20 wt.% Pt/C catalyst and high stability during the 120 h test. This work provides a groundbreaking pathway for the large‐scale production of high single‐atom areal density, thermally robust SACs, advancing their practical applications in clean energy technologies.

## Graphical abstract

This article demonstrates a vacuum direct current arc discharge strategy for synthesizing Pt single‐atom catalysts (SACs) with an ultra‐high Pt areal density. The resulting catalyst shows remarkable thermal stability at 1000 °C and excellent performance in water splitting. This work provides a scalable and facile approach for producing high‐density, thermally robust Pt‐group SACs for clean energy applications.

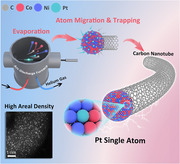

## 1 Introduction

Over the last decade, single‐atom catalysts (SACs) have been receiving increased attention for the platinum (Pt) group metal–based thermal‐ and electro‐catalytical applications,^[^ ^1^ ^]^ offering maximal utilization and control of the catalytic activity at the atomic level.^[^ ^2^, ^3^, ^4^ ^]^ To further advance the structure and performance of the SACs, one option is an appropriate selection and precise design of the support and/or promoter.^[^ ^5^, ^6^ ^]^ For instance, through the design of a Cu/Pt single‐atom alloy, the C─H bond activation efficiency and coke resistance were reported to be improved remarkably.^[^ ^7^ ^]^ Another option is to increase the metal loading. Nevertheless, due to the Gibbs–Thomson effect, single atoms tend to be aggregated under high metal loading.^[^ ^8^ ^]^ Accordingly, most of the researches are limited to a maximum loading of 2 wt.% for the synthesis of SACs, and the metal areal density of most catalysts is under 6.5 atoms nm^−2^.^[^ ^9^, ^10^, ^11^, ^12^ ^]^ Larger metal mass loading and areal density for SACs are significant in this field, but also remain challenging to achieve.^[^ ^13^ ^]^

More critically, the development of large‐scale synthesis strategies (e.g., gram‐ or kilogram‐scale) is urgently needed in promoting the practical application and commercialization of SACs.^[^ ^14^ ^]^ To date, the existing methods, encompassing both top–down approaches (i.e., thermochemical and electrochemical) and bottom–up methods (i.e., wet chemical processes, pyrolysis, and atomic layer deposition),^[^ ^5^, ^15^ ^]^ are limited to milligram‐scale production, restricting their applicability to laboratory testing. Furthermore, these methods are often complex and involve multiple steps including downstream solvent removal/recovery and product modification.^[^ ^16^, ^17^, ^18^ ^]^ Recently, a variety of emerging strategies, including the metal coordination strategy^[^ ^19^ ^]^ and solvothermal method,^[^ ^20^ ^]^ have been trialed with success either in achieving kilogram‐scale product yield or one‐pot synthesis of SACs. Nevertheless, these approaches are still far from mature enough in demonstrating a high commercial potential which ideally needs to involve both single‐step and mass production.^[^ ^21^, ^22^, ^23^ ^]^

**Figure 1:**
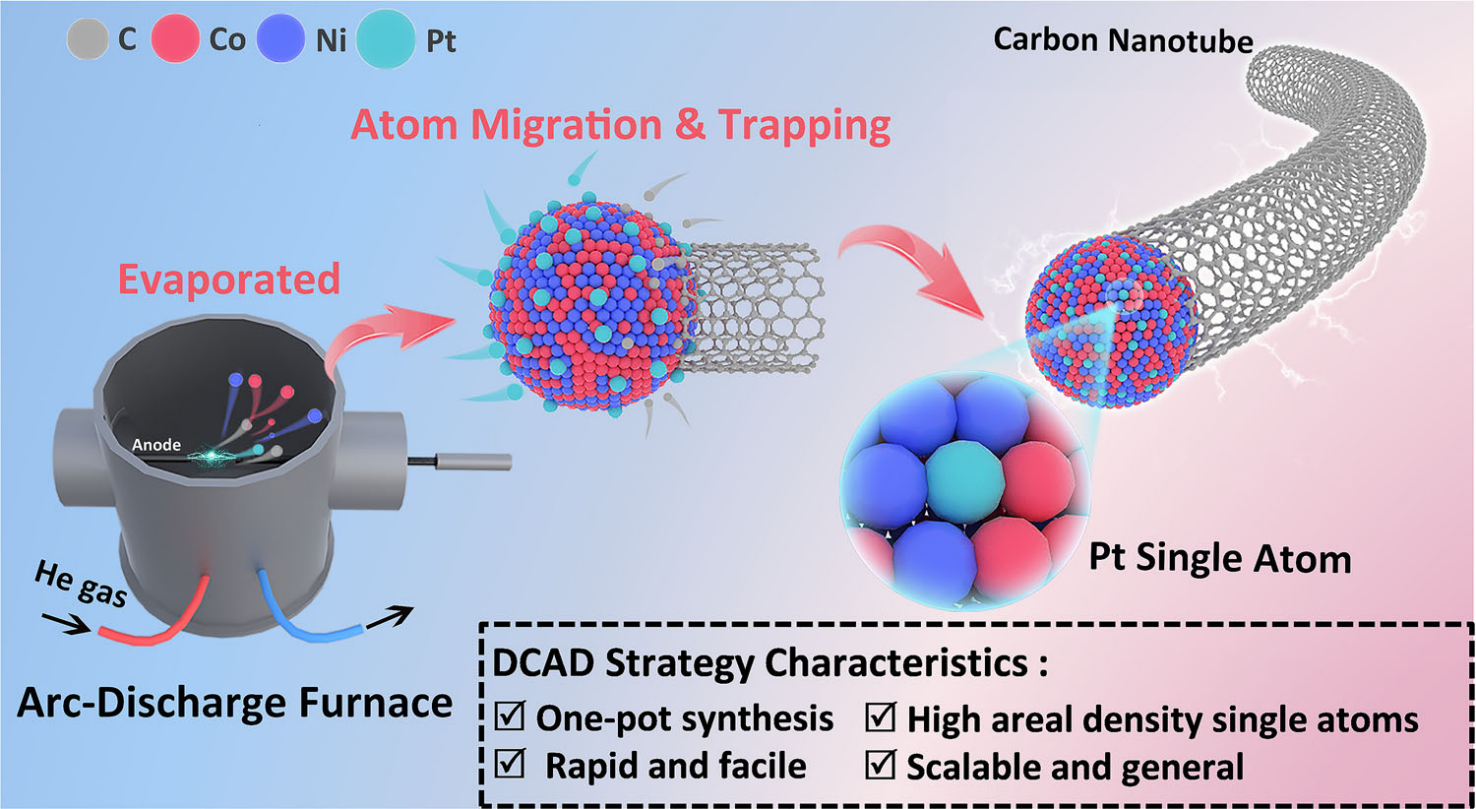
Schematic diagram of SACs synthesis via the DCAD method.

Herein, we report a facile, one‐pot synthesis approach for unprecedentedly high loading of ≈4 wt.% Pt SAC with an ultra‐high Pt areal density of 10.6 atom nm^−2^. As illustrated in **Figure** **1**, our strategy is simple, and its core approach is a commercially available vacuum direct current arc discharge (DCAD) method, which is alike the existing flash thermal‐quenching procedure,^[^ ^24^ ^]^ while superior in offering an estimated core temperature of 4000–6000 K that is sufficient for all the elements to vaporize. Subsequently, the resultant atomic vapor quickly migrates and re‐assembles at a quenching rate of ≈166 K ms^−1^ on a cooling water‐jacketed chamber wall.^[^ ^25^ ^]^ The precursors are also simple, including metallic cobalt (Co), nickel (Ni), Pt oxide, and graphite that are physically mixed beforehand. The former two metals are expected to vaporize rapidly and condense into an alloy that can subsequently exert a trapping effect in stabilizing the Pt single‐atoms, forming a stable binding that can remain intact at the extremely high temperature created by the arc discharge facility, which, in turn, is expected to maintain high stability for the Pt single‐atoms.^[^ ^26^ ^]^ Furthermore, the CoNi alloys are expected to catalyze the atomized carbon into carbon nanotubes (CNTs).^[^ ^27^ ^]^ The resultant CNTs can then serve as a robust matrix to promote the electronic conductivity for electrocatalysis, but also provide spatial confinement in hosting and isolating the metals inside its nanometer‐wide individual cavities.^[^ ^28^ ^]^ We further demonstrated its universality in extending to other noble metal SAC synthesis such as iridium (Ir). Additionally, through two probe reactions including electrocatalytic hydrogen evolution reaction (HER) and oxygen evolution reaction (OER), we demonstrated the superiority of the catalyst (CoNiPt_SA_@G). In HER, it enables a low overpotential at 10 mA cm^−2^ of 23 mV, a high mass activity of 13.25 A mg_Pt_^−1^ at 300 mV that is more than six times of the commercial 20 wt.% Pt/C catalyst, and a high stability under 120 h operation with trivial dissolution of Pt into electrolyte (0.67 µg L^−1^). In OER, CoNiPt_SA_@G exhibits a low overpotential of 213 mV at 10 mA cm^‐^^2^, which is 74 mV lower than that of RuO_2_ catalyst. Collectively, these attributes establish DCAD as a facile, scalable, and versatile platform that holds significant promise for advancing the practical applications of SACs to clean energy and sustainable processing in a carbon‐constrained world.

## 2 Synthesis and Characterization

The synthesis procedure for the CoNiPt_SA_@G catalyst is detailed in the Experimental Section (Supporting Information). In brief, for a typical batch synthesis, the metallic Co powder, Ni powder, PtO_2_ powder, and graphite powder were mixed and filled into a customized hollow graphite rod. The filled rod was assembled as the anode in the DCAD chamber, where a pure graphite rod was used as the cathode. A stable arc was discharged between the anode and cathode, creating a high core temperature of up to 6000 K that can lead to the immediate evaporation of the anode. The metallic Co and Ni have the lowest evaporation temperatures and fastest diffusion rates, and hence, they are expected to evaporate first, diffuse to and condense into CoNi nano alloy on the cooling wall. Subsequently, the atomized Pt, once migrating to the cooling wall, is trapped by the previously formed CoNi alloy. Furthermore, due to the catalytic effect of CoNi alloy, the evaporated C atoms are expected to grow into CNTs. A single batch usually lasts less than 30 min until the anode is fully burnt out. Afterward, the arc was terminated, and the condensed products were collected from the wall surface. For comparison, the other four reference samples were synthesized, including Pt@G with the absence of Co and Ni, CoPt@G and NiPt@G for the co‐presence of either Ni or Co with Pt, and CoNi@G with the absence of Pt.

Elemental compositions determined by inductively coupled plasma‐optical emission spectroscopy (ICP‐OES) in Figure S1a (Supporting Information) confirmed an identical loading of Pt of ≈4 wt.% across the four samples. So are the contents of Co and Ni in CoNi@G and CoNiPt_SA_@G which also agree with the amounts expected (Figure S1b, Supporting Information). This method demonstrates high productivity, completing a single batch within 30 min and achieving gram‐level yields. The platinum utilization was calculated to be 72.15% (Table S1, Supporting Information), with losses primarily from the metal deposits on the cathode caused by electric field‐driven metal ion migration and, to a lesser extent, from the carbon soot collection process (Figure S1c, Supporting Information). Figure S1d and Table S2 (Supporting Information) for the materials cost analysis reveal that the cost per unit of catalyst synthesized via DCAD is only US$4.6 g^−1^, significantly lower than that of 20 wt.% Pt/C (US$68.7 g^−1^) and RuO_2_ catalysts (US$38.6 g^−1^).

**Figure 2:**
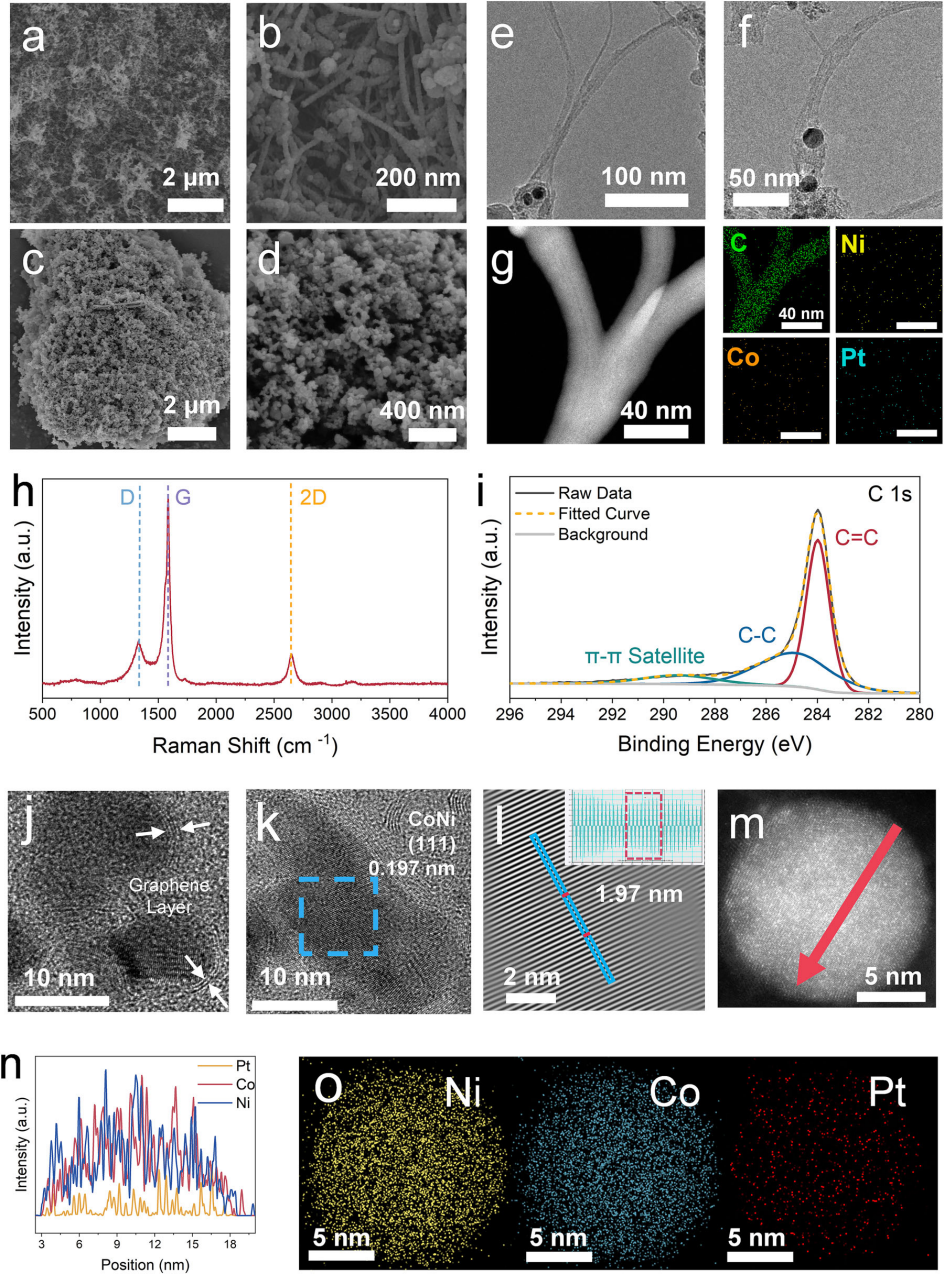
a,b) SEM images of CoNiPt_SA_@G. c,d) SEM images of Pt@G. e,f) TEM images of the CNTs structure in CoNiPt_SA_@G. g) HAADF‐TEM image of the CNTs structure in CoNiPt_SA_@G and the corresponding elemental mapping results. h) Raman spectra of CoNiPt_SA_@G. i) XPS C_1s_ spectra of CoNiPt_SA_@G. j,k) HR‐TEM image of CoNiP_tSA_@G. l) The lattice space of the corresponding area in panel k. m,n), Elemental intensities for the Pt L‐edge, Ni K‐edge, and Co K‐edge (n) along the scanned red arrow as indicated in the HAADF‐STEM image (m). o) HAADF‐STEM EDS elemental mapping of scanned area (m) of CoNiPt_SA_@G.

Furthermore, the detailed structural characterization confirmed our hypothesis on the desired formation of CNTs and their encapsulation of Pt single‐atoms within the CoNiPt_SA_@G catalyst. **Figure** **2**a,b for the scanning electron microscope (SEM) images demonstrate the abundance of a fibrous structure that is attached with visible beads, relative to the predominance of round‐shaped structures for the Pt@G reference (Figure 2c,d). Figure S2a (Supporting Information) confirmed a mean size of ≈15 nm for the fibrous structure within CoNiPt_SA_@G, which is large enough to confine these metal particles, as the majority of particles are less than 10 nm in size (Figure S2b,c, Supporting Information). Indeed, the transmission electron microscope (TEM) observations in Figure 2e,f and Figure S3 (Supporting Information) confirmed a preferred encapsulation of metal nanoparticles at one end of the CNTs. The scanning TEM (STEM) elemental mapping in high‐angle annular dark field (HAADF) mode (Figure 2g) also confirmed that the fibers are exclusively composed of carbon. In addition, as shown in Figure S4 (Supporting Information), the fibrous structure is absent for the other two reference samples of CoPt@G and NiPt@G, demonstrating the essentiality of the CoNi alloy in the formation of CNTs from arc discharge.

Figure 2h for the Raman spectra of CoNiPt_SA_@G catalyst confirmed a high graphitization of the carbon support, demonstrating a typical CNT structure with three distinct peaks located at ≈1330 cm^−1^ for D band, 1580 cm^−1^ for G band and 2650 cm^−1^ for 2D band, and an *I*_D_/*I*_G_ intensity ratio of 0.30 (Table S3, Supporting Information) that aligns closely with multiple‐walled CNTs.^[^ ^29^ ^]^ Likewise, the X‐ray photoelectron spectroscopy (XPS) C_1s_ spectra of CoNiPt_SA_@G (Figure 2i) can be deconvoluted into three peaks, 284 eV (C═C), 284.8 eV (C─C), and 289.4 eV (*π–π* satellite) that align with the typical CNTs too.^[^ ^30^ ^]^ In the DCAD process, the formation of CNTs proceeds via a tip‐growth mechanism. Under the intense arc plasma, graphite powder is rapidly vaporized, releasing carbon atoms into the gas phase. These carbon atoms dissolve into the CoNi alloy nanoparticles under the high‐temperature arc. Upon reaching saturation, carbon precipitates and reorganizes into sp^2^‐hybridized graphitic layers, forming tubular walls of the CNTs. The continuous incorporation of carbon leads to the elongation of the nanotube, while the metal particle remains at the tip of the growing CNTs.^[^ ^31^, ^32^ ^]^ The resultant CNTs form a conductive and robust support for metal nanoparticles, which can accelerate the electron transfer during the electrocatalysis.^[^ ^33^, ^34^ ^]^ The high‐resolution TEM (HR‐TEM) image in Figure 2j further confirmed that these Co and Ni nano particles are spatially confined by 2–3 layers of graphene, with a featured inter‐planar distance of 0.197 nm for the (111) plane of CoNi alloy amplified in Figure 2k,l. Notably, linear scanning and elemental mapping (Figure 2m–o) under HAADF mode demonstrate that Pt is effectively anchored on the CoNi substrate, confirming the effective trapping of Pt by the CoNi nanoalloy.

**Figure 3:**
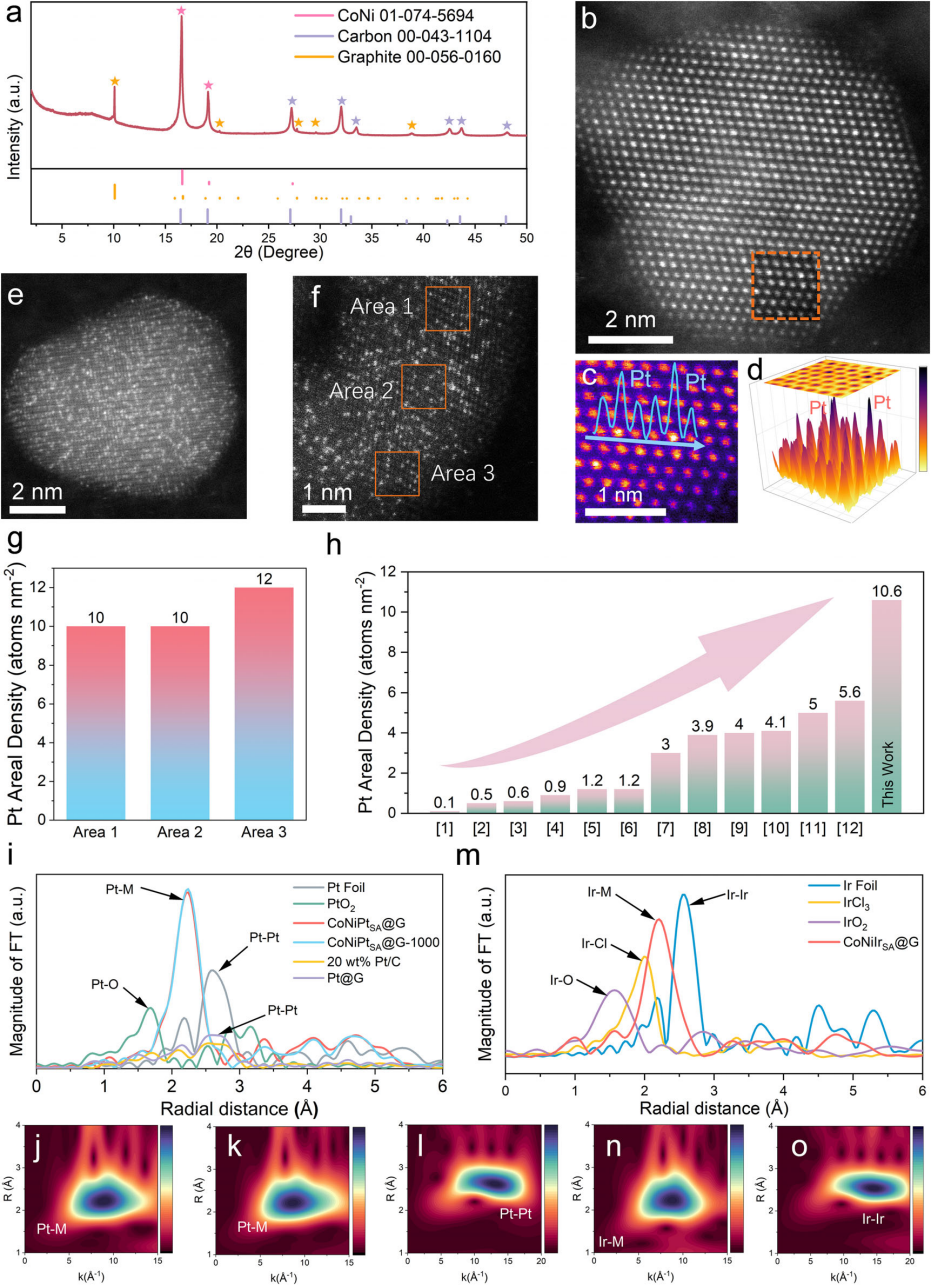
a) synchrotron PD pattern of CoNiPt_SA_@G. b) AC‐HAADF‐STEM image of CoNiPt_SA_@G. c) An enlarged section of the orange box in (b). The blue curve was the integrated pixel intensity in the marked box. d) 3D surface plot of the marked box in (b). e,f AC‐HAADF‐STEM images of CoNiPt_SA_@G. g, Statistic pixel analysis of the Pt atom areal density in three areas in (f). h) Comparison of the Pt areal density of SACs in this work and previously reported literature. i) Pt L_3_ edge FT‐EXAFS spectra in R space of CoNiPt_SA_@G and CoNiPt_SA_@G‐1000 along with Pt foil and PtO_2_ references. j–l) Wavelet transform (WT) contour map for the k^3^‐weighted EXAFS signal of CoNiPt_SA_@G (j), CoNiPt_SA_@G‐1000 (k), and Pt foil (l). m) Ir L_3_ edge FT‐EXAFS spectra in R space of CoNiIr_SA_@G, Ir foil, IrCl_3_ and IrO_2_ references. n,o) Wavelet transform (WT) contour map for the k^3^‐weighted EXAFS signal of CoNiIr_SA_@G (n) and Ir foil (o).

**Figure** **3**a shows the high‐resolution synchrotron powder diffraction (PD) pattern of the CoNiPt_SA_@G catalyst. The principal diffraction peaks can be indexed as CoNi alloy (PDF 01‐074‐5694) and carbon phase (graphite PDF 00–056‐0160 and carbon PDF 00‐043‐1104), whilst the Pt‐bearing phase is non‐detectable, indicative of its atomic dispersion. In contrast, the metallic Pt^0^ (PDF 03‐065‐2868) was found in Pt@G (Figure S5a, Supporting Information). These results further verified the critical role of CoNi nano alloy in trapping and isolating Pt atoms. The diffraction peaks (Figure S5b, Supporting Information) for CoNi@G coincide well with CoNi alloy; and the remaining two references with a single transition metal (CoPt@G and NiPt@G) show the abundance of metallic Co (PDF 00‐001‐1278, PDF 00‐015‐0806) and Ni (PDF 00‐001‐1258) (Figure S5c,d, Supporting Information), as expected. Nevertheless, the Pt‐bearing peaks are still invisible in these two samples, demonstrating a promotional role of the two transition metals on the atomic dispersion of Pt. Moreover, the hydrogen adsorption/desorption profiles of CoNiPt_SA_@G and the commercial 20 wt.% Pt/C reference, observed from the cyclic voltammetry (CV) curves in Figure S6 (Supporting Information), further supports an atomic dispersion of Pt in the former catalyst. The peaks associated with underpotentially deposited hydrogen (H_upd_) appear at 0.3 and 0.4 V for 20 wt.% Pt/C. In contrast, these H_upd_ peaks for CoNiPt_SA_@G are invisible, which is a key feature of the Pt single‐atoms.^[^ ^35^, ^36^ ^]^

By using the dint of spherical aberration corrected TEM (AC‐TEM), Pt was also visually confirmed to exist as single atoms that are featured by the appearance of discrete bright dots which are individually dispersed on the CoNi alloy (Figure 3b). Indeed, the atomic dispersion of Pt in CoNiPt_SA_@G was also verified by the integrated pixel intensity profile and 3D surface plot in Figure 3c,d. For the reference Pt@G catalyst, HR‐TEM (Figure S7a,b, Supporting Information) images confirmed the presence of a lattice fringe of metallic Pt (111) plane with an inter‐planar distance of 0.21 nm, whereas the fraction of Pt as single‐atoms is much lower (marked in the red circle, Figure S7c, Supporting Information). Here again, these notable differences underscore the essentiality of Co and Ni in trapping Pt as individual single atoms. The AC‐HAADF‐STEM image of CoNiPt_SA_@G (Figure 3e; Figure S8a, Supporting Information) reveals an exceptionally high areal density of uniformly dispersed Pt single atoms which are present as distinct bright dots. Pixel statistical analysis across three regions marked in Figure 3f confirms an average Pt areal density of 10.6 atoms nm^−2^ (Figure 3g), a remarkable value considering that most reported Pt SACs exhibit areal densities below 5.6 atoms nm^−2^ (Figure 3h, Table S4, Supporting Information). Moreover, the average areal density of Pt atoms was calculated by incorporating the Brunauer‐Emmett‐Teller (BET) surface area into the calculation.^[^ ^9^ ^]^ For CoNiPt_SA_@G, the BET surface area was measured to be 76 m^2^ g^−1^ (Figure S8b, Supporting Information), and the average areal density of Pt was calculated to be 1.5 atoms nm^−2^, which still surpasses many previously reported Pt SAs catalysts (Table S5, Supporting Information).

Figure 3i exhibits the respective Fourier‐transformed (FT) extended X‐ray absorption fine structure (EXAFS) profile in R space of Pt L_3_ edge in the catalysts and Pt standards. The spectrum of CoNiPt_SA_@G is distinct in having only one peak located at 2.24 Å, indicative of the lack of Pt in an aggregate form.^[^ ^37^, ^38^ ^]^ This peak can be assigned to the coordination of Pt─*M* where *M* is for Ni or Co. In contrast, the R space EXAFS spectrum of the Pt@G sample closely resembles that of the commercial 20 wt.% Pt/C catalyst, showing a characteristic Pt–Pt coordination peak for Pt clusters at ≈2.59 Å. These findings highlight the critical role of the CoNi alloy in effectively isolating Pt atoms and preventing their aggregation. To testify the hypothesis on the extreme stability of our Pt single atoms that were prepared by the high‐temperature arc discharging approach, we further annealed the CoNiPt_SA_@G at 1000 °C for 2 h under N_2_ atmosphere (denoted as CoNiPt_SA_@G‐1000). Its respective EXAFS profile (Figure 3i) also shows a single peak at ≈2.24 Å. In addition, the k^2^‐weighted Pt L_3_‐edge EXAFS oscillations of CoNiPt_SA_@G and CoNiPt_SA_@G‐1000 in k space show negligible difference (Figure S9a , Supporting Information), indicating Pt SAs remain intact after high‐temperature annealing. Such stability is outstanding for the ultra‐high areal density of Pt loaded in the catalyst. Moreover, the wavelet transform (WT) patterns of the k^3^‐weighted EXAFS oscillations for both CoNiPt_SA_ @G and CoNiPt_SA_@G‐1000 (Figure 3j,k) show a prominent intensity located at *R* ≈ 2.2–2.3 Å and *k* ≈ 7.5 Å^−1^ for Pt–M coordination, which are distinct from the WT patterns of standard Pt foil and PtO_2_ (Figure 3l; Figure S9b, Supporting Information). Figure S9c (Supporting Information) for the WT pattern of Pt@G exhibits the strong Pt–Pt coordination. Noteworthily, the DCAD strategy was also successfully applied to Ir for high dispersion of this metal as single atoms within CoNiIr_SA_@G, as evidenced by the EXAFS spectra in R space of CoNiIr_SA_@G and Ir‐related standards in Figure 3m where a single peak located at 2.17 Å appeared. This feature applies to the first shell of the Ir–M (M: Co/Ni) coordination.^[^ ^39^ ^]^ Additionally, Figure 3n for the WT contour map of CoNiIr_SA_@G confirms the strong Ir–M coordination. Its main peak locates at *R* ≈ 2.17 Å and *k* ≈ 8.5 Å^−1^ that is distinct from the Ir–Ir, Ir–O, and Ir–Cl coordination shown in Figure 3o, Figure S9d,e (Supporting Information), respectively.

**Figure 4:**
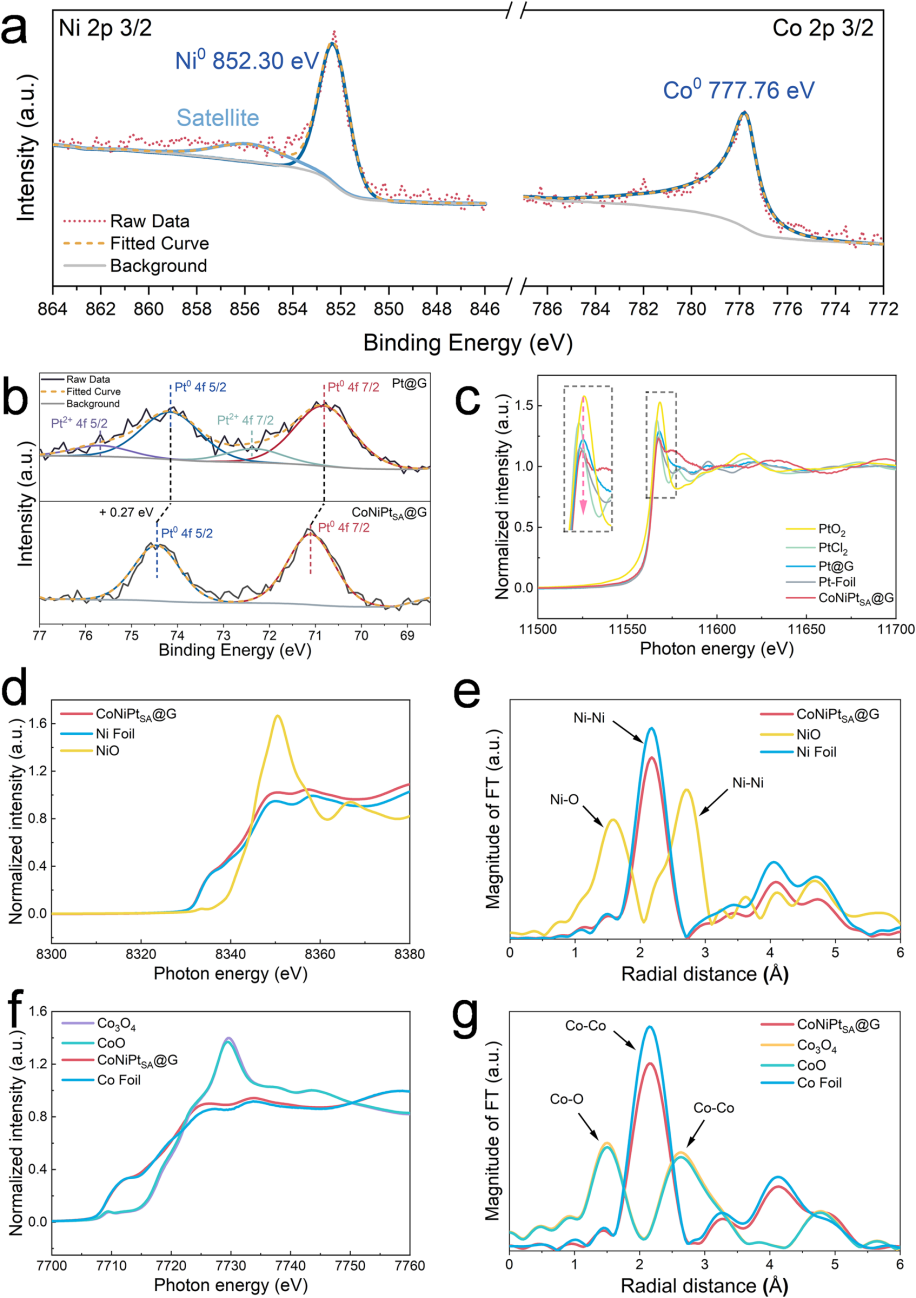
a) Co 2p and Ni 2p high‐resolution XPS spectra of CoNiPt_SA_@G. b) Pt 4f high‐resolution XPS spectra of Pt@G and CoNiPt_SA_@G. c) Pt L_3_ edge normalized XANES spectra of CoNiPt_SA_@G and Pt@G along with PtCl_2_, PtO_2_, and Pt foil as standards. d) Ni K edge normalized XANES of CoNiPt_SA_@G along with NiO and Pt foil as standards. e) Ni K edge FT‐EXAFS spectra in R space of CoNiPt_SA_@G along with Ni foil and NiO. f) Co K edge normalized XANES spectra of CoNiPt_SA_@G along with CoO, Co_3_O_4_, and Co foil as standards. g) Co K edge FT‐EXAFS spectra in R space of CoNiPt_SA_@G along with CoO, Co_3_O_4_, and Co foil.

The electronic structure of catalysts was investigated by XPS. As shown in **Figure** **4**a, the peaks of Co 2p 3/2 and Ni 2p 3/2 for CoNiPt_SA_@G are respectively located at 777.76 and 852.3 eV, which can be attributed to the metallic state.^[^ ^40^, ^41^ ^]^ For other catalysts from CoNi@G to NiPt@G and CoPt@G, the valence states of Co and Ni are metallic as well (Figure S10a–c, Supporting Information). Figure 4b is the Pt 4f spectra of Pt@G and CoNiPt_SA_@G. The former catalyst has two doublets in its deconvoluted spectrum. The first doublet is located at 70.83 and 74.18 eV for Pt^0^ 4f/2 and 4f5/2, respectively. Another doublet is at 72.33 and 75.68 eV, which can be attributed to Pt^2+^ 4f/2 and 4f5/2,^[^ ^42^ ^]^ respectively. In contrast, for the latter catalyst of CoNiPt_SA_@G, a single doublet with two peaks located at 71.1 and 74.45 eV was observed, which can be assigned to the 4f7/2 and 4f5/2 orbitals of Pt^0^, respectively. Moreover, both peaks have a blueshift of ≈0.27 eV as compared to the corresponding peaks in the spectrum of Pt@G (70.83 and 74.18 eV). This is caused by the electronic metal–support interaction between Pt single atoms and CoNi support.^[^ ^43^, ^44^ ^]^ However, the binding energy of Pt^0^ in CoNiPt_SA_@G is still lower than that of standard metallic Pt provided in the Avantage Knowledge Base, indicating an electron‐rich nature of Pt in CoNiPt_SA_@G. A similar interaction was also observed in the XPS spectra of CoPt@G and NiPt@G in Figure S11a and Table S6 (Supporting Information). Additionally, regarding the Pt L3‐edge XANES spectra in Figure 4c, the white‐line intensity of CoNiPt_SA_@G is slightly lower than that of the Pt foil standard, indicating the presence of a negatively charged Pt state.^[^ ^45^ ^]^ In contrast, the white‐line intensity of Pt@G lies between that of Pt foil and PtCl_2_, suggesting a positively charged Pt state in this sample.^[^ ^46^ ^]^ Based on the fitting of the white‐line intensity from standard samples (Figure S11b, Supporting Information), the oxidation valency of Pt in CoNiPt_SA_@G and Pt@G is estimated to be −0.54 and +0.63, respectively, which is in good agreement with the findings from the XPS analysis.

The Ni K edge XANES spectra of CoNiPt_SA_@G are illustrated in Figure 4d,e. The metallic Ni^0^ is abundant, with a predominant radial distance of 2.18 Å that can be assigned to the first‐shell Ni–Ni coordination.^[^ ^47^, ^48^ ^]^ Similarly, the XANES at the Co K edge in Figure 4f indicates the abundance of metallic state, whilst Figure 4g for the respective FT‐EXAFS at R space supports the predominance of Co─Co bonding in the first shell.^[^ ^49^ ^]^ The reduced intensities of the first‐shell coordination peaks in both EXAFS spectra of Co and Ni for CoNiPt_SA_@G (compared to pure Co and Ni foil standards) indicate the formation of a CoNi nanoalloy with solid solution characteristics.^[^ ^50^ ^]^ This intensity reduction arises from the atomic‐level mixing of Co and Ni atoms, which introduces structural disorder and modifies the local coordination environments. Furthermore, the Ni and Co K‐edge EXAFS spectra of CoNiPt_SA_@G reveal additional coordination shells at radial distances of 4.05 and 4.7 Å (Ni K edge) and 3.28 and 4.14 Å (Co K edge), respectively. These characteristic distances provide clear evidence of a face‐centered cubic (fcc) crystalline structure.^[^ ^51^ ^]^

## 3 Electrocatalytic Performance Evaluation

**Figure 5:**
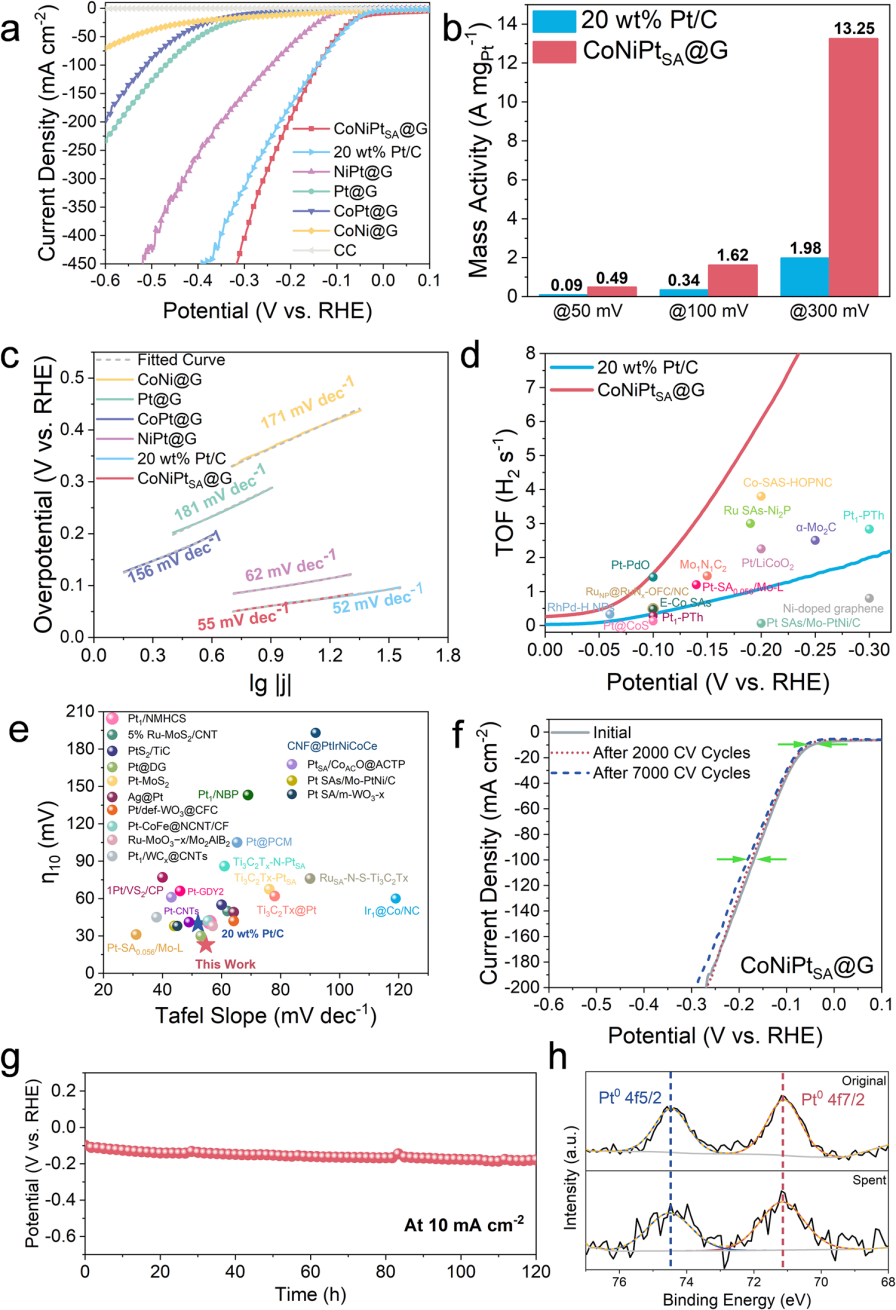
HER Performance in Acidic Media. a) Polarization curves of CoNiPt_SA_@G and other reference catalysts and commercial 20 wt.% Pt/C catalyst in the 0.5 m H_2_SO_4_ electrolyte after 90% i‐R compensation. b) Mass activity normalized by the Pt loading of CoNiPt_SA_@G and 20 wt.% Pt/C at different potentials. c) Tafel slopes of CoNiPt_SA_@G and reference catalysts. d) TOF values for CoNiPt_SA_@G, 20 wt.% Pt/C, and other reported electrocatalysts over a wide range of overpotentials. e) Comparison of overpotentials at 10 mA cm^−2^ and Tafel slopes of CoNiPt_SA_@G with other reported HER catalysts in acidic electrolytes. f) Polarization curves for CoNiPt_SA_@G before and after 7000 catalytic cycles in 0.5 m H_2_SO_4_ electrolyte. g) Chronopotentiometry test of CoNiPt_SA_@G at a current density of 10 mA cm^−2^. h) XPS Pt 4f Spectra of CoNiPt_SA_@G before and after Stability Test.

In this study, we use the HER as a primary probe reaction to demonstrate the potential application of our strategy in electrocatalysis. The HER catalytic performance was evaluated in a three‐electrode system in N_2_ – saturated 0.5 m H_2_SO_4_ electrolyte. **Figure** **5**a shows the linear sweep voltammetry (LSV) curves of as‐synthesized samples, commercial 20 wt.% Pt/C reference and pure carbon cloth (denoted as CC hereafter). Clearly, the CoNiPt_SA_@G catalyst is superior in exhibiting the highest apparent activity, along with the least overpotential and highest current density (normalized by geometric area) within the potential range. For instance, at the current density of 10 mA cm^−2^ (η_10_), the overpotential of CoNiPt‐1@G reaches 23 mV, which is lower than the values of 39 mV for the 20 wt.% Pt/C reference (Figure S12a, Supporting Information). Additionally, the metal‐free CC lacks activity, whilst the CoNi@G without Pt is much less active than those with Pt, substantiating the predominance of Pt as the active site. By normalizing the measured current based on the Pt content, Figure 5b displays the mass activity of CoNiPt_SA_@G and 20 wt.% Pt/C catalysts at three different potentials. Quantitatively, the CoNiPt_SA_@G catalyst is superior in having a mass activity that is more than five times larger than the 20 wt.% Pt/C reference, echoing the significance of the single‐atom dispersion of Pt within the CoNiPt_SA_@G synthesized by our unique method.

Figure 5c shows the Tafel plots derived from the polarization curves. Among all the as‐synthesized catalysts, CoNiPt_SA_@G has the least Tafel slope of 55 mV dec^−1^, which is also very close to the 20 wt.% Pt/C catalyst with a Tafel slope of 52 mV dec^−1^. Furthermore, the exchange current density (*j*_0_) was estimated by extrapolating the linear region of Tafel plots back to zero overpotential to indicate the kinetics of each electrocatalyst.^[^ ^52^ ^]^ The *j*_0_ of CoNiPt_SA_@G was calculated to be 0.61 mA cm^−2^, surpassing the commercial 20 wt.% Pt/C of 0.53 mA cm^−2^ and all the other reference catalysts ranging from 0.06 to 0.23 mA cm^−2^. All these results are further summarized in Table S7 (Supporting Information).

Another comparison is the intrinsic HER catalytic activity that can be quantified by the electrochemically active surface area (ECSA) which is estimated from the measurement of the double‐layer capacitance (*C*_dl_) via CV curves (Figures S12b,S13, Supporting Information).^[^ ^53^, ^54^ ^]^ As demonstrated in Figure S12c (Supporting Information), CoNiPt_SA_@G still outperforms all the other counterparts in reaching the largest current within the potential range. Specifically, at the overpotential of 0.35 V, the current density of CoNiPt_SA_@G reaches 0.5 mA cm_ECSA_^−2^ while the 20 wt.% Pt/C reference has a current density of less than 0.35 mA cm_ECSA_^−2^. Additionally, another activity indicator, electron conductivity, was probed by electrochemical impedance spectroscopy (EIS) and is displayed in the Nyquist plot (Figure S12d, Supporting Information).^[^ ^55^ ^]^ The charge transfer resistance (*R*_ct_) was obtained by fitting the Nyquist plot per the equivalent circuit. Here again, among all the as‐prepared catalysts, the CoNiPt_SA_@G one exhibits the least *R*_ct_ value of 8 Ω, followed by NiPt@G with an *R*_ct_ of 34 Ω, and the remainder exceeding 100 Ω. This result indicates that the well‐developed CNT network within the CoNiPt_SA_@G significantly enhances electrical conductivity, thereby accelerating interfacial electron transfer and facilitating a faster Faradaic process at the catalyst‐electrolyte interface.^[^ ^56^, ^57^, ^58^ ^]^

Figure 5d plots the TOF profiles of CoNiPt_SA_@G and commercial Pt/C as a function of the potential, with the inclusion of the literature‐reported catalysts for comparison. The results are also tabulated in Table S8 (Supporting Information). Apart from suppressing the 20 wt.% Pt/C reference for any given potential, the CoNiPt_SA_@G catalyst is also comparable to or even superior over the reported ones. Likewise, in terms of overpotential and Tafel slope in Figure 5e and Table S9 (Supporting Information), the HER performance of CoNiPt_SA_@G catalyst is also better than most of the noble metals and SACs reported in acidic electrolytes. Additionally, we tested the catalytic performance of the CoNiIr_SA_@G catalyst. As shown in Figure S14a,b (Supporting Information), CoNiIr_SA_@G exhibited a *η*_10_ of 50 mV and a low Tafel slope of 38 mV dec^−1^, and surpassed the performance of 20 wt.% Pt/C catalyst at high current densities. These results further demonstrate the universality of our strategy.

Finally, the CoNiPt_SA_@G catalyst was tested for its durability by several different measures. For the LSV curves collected after 7000 CV sweeps in Figure 5f, the shift is very much negligible after the 2000 CV sweeps and trivial after the 7000 CV sweeps. More strikingly, Figure 5g and Figure S15 (Supporting Information) for the chronopotentiometry (CP) measurement at 10 and 30 mA cm^−2^ substantiate a strong durability of CoNiPt_SA_@G with only a small potential drop of 80 mV at 10 mA cm^−2^ after 120 h and 78 mV at 30 mA cm^−2^ after 100 h. Furthermore, the post‐CP test electrolyte was analyzed by ICP‐OES, revealing a trivial loss of Pt for an extremely low concentration of 0.67 µg L^−1^ within the electrolyte after 120 h test (Table S10, Supporting Information). Figure S16a,b (Supporting Information) are the SEM images of the spent CoNiPt_SA_@G after a 120 h stability test, where the fibrous structure of CNTs remains clearly visible. In Figure S16c,d (Supporting Information), the TEM images of the spent catalyst also reveal a structure similar to that of the fresh sample. In comparison to the fresh sample, the Pt 4f XPS spectra of the spent CoNiPt_SA_@G in Figure 5h show no peak shift, confirming the structural integrity of the Pt SA sites after prolonged testing. Moreover, the (111) plane of CoNi was also confirmed in the HR‐TEM images and the corresponding lattice spacing pattern of the metal particles (Figure S16e,f, Supporting Information), indicating that the CoNi alloy substrate retains its structural integrity during the HER process.

The CoNiPt_SA_@G catalyst was also tested for oxygen evolution reaction (OER) in an O_2_‐saturated 1 m KOH electrolyte. Figure S17a (Supporting Information) shows the polarization curves of all the as‐synthesized electrocatalysts and the commercial RuO_2_ catalyst. Evidently, CoNiPt_SA_@G requires the least overpotential of 213 mV to reach an apparent current density of 10 mA cm^−2^, which is 74 mV lower than that of the RuO_2_ catalyst (Table S11, Supporting Information). More intriguingly, for the CoNi@G catalyst, it achieved a *η*_10_ value of 503 mV that is more than double of the CoNiPt_SA_@G. This further substantiates a significant and irreplaceable role of Pt single‐atom, although its bulk content is merely 3.82 wt.% within CoNiPt_SA_@G. Figure S17b (Supporting Information) for the Tafel slopes also confirmed a least slope of 148 mV dec^−1^ for the CoNiPt_SA_@G among all the catalysts. We also calculated the TOF values of CoNiPt_SA_@G and RuO_2_ (Figure S17c, Supporting Information). The TOF of CoNiPt_SA_@G reaches 1.72 × 10^−1^ O_2_ s^−1^ at 1.53 V versus reversible hydrogen electrode (RHE), which is two orders of magnitude larger than that of RuO_2_ (1.64 × 10^−3^ O_2_ s^−1^). More strikingly, the TOF of CoNiPt_SA_@G is larger than the recently reported OER catalysts including Ir and Ru‐based ones (Table S12, Supporting Information). Nyquist plots obtained from EIS manifest that the CoNiPt_SA_@G catalyst has the smallest *R*_ct_ (27 Ω) compared with others, suggesting its fast charge transfer at the interface (Figure S17d, Table S11, Supporting Information). Furthermore, the overall water splitting performance of the CoNiPt_SA_@G catalyst was evaluated in a two‐electrode configuration, as either the cathode (−) or the anode (+) in 0.5 m H_2_SO_4_ and 1 m KOH electrolytes, respectively. IrO_2_ and Pt/C were used as benchmark references. Figure S18a (Supporting Information) for the LSV curves of the overall waters splitting in 0.5 m H_2_SO_4_ reveals that the assembly of IrO_2_ (+) || CoNiPt_SA_@G (−) requires a cell voltage of 1.66 V to achieve a current density of 10 mA cm^−2^, which is lower than the 1.7 V required by the bench mark IrO_2_ (+) || 20 wt.% Pt/C (−) assembly. Similarly, as shown in Figure S18b (Supporting Information), the assembly of CoNiPt_SA_@G (+) || 20 wt.% Pt/C (−) reaches 10 mA cm^−2^ at a cell voltage of 1.65V in 1 m KOH electrolyte, outperforming the IrO_2_ (+) || 20 wt.% Pt/C (−) configuration which requires a cell voltage of 1.71 V. These results highlight the promising potential of CoNiPt_SA_@G as an efficient bifunctional electrocatalyst for overall water splitting.

## 4 Mechanistic Study and Theoretical Calculation

**Figure 6:**
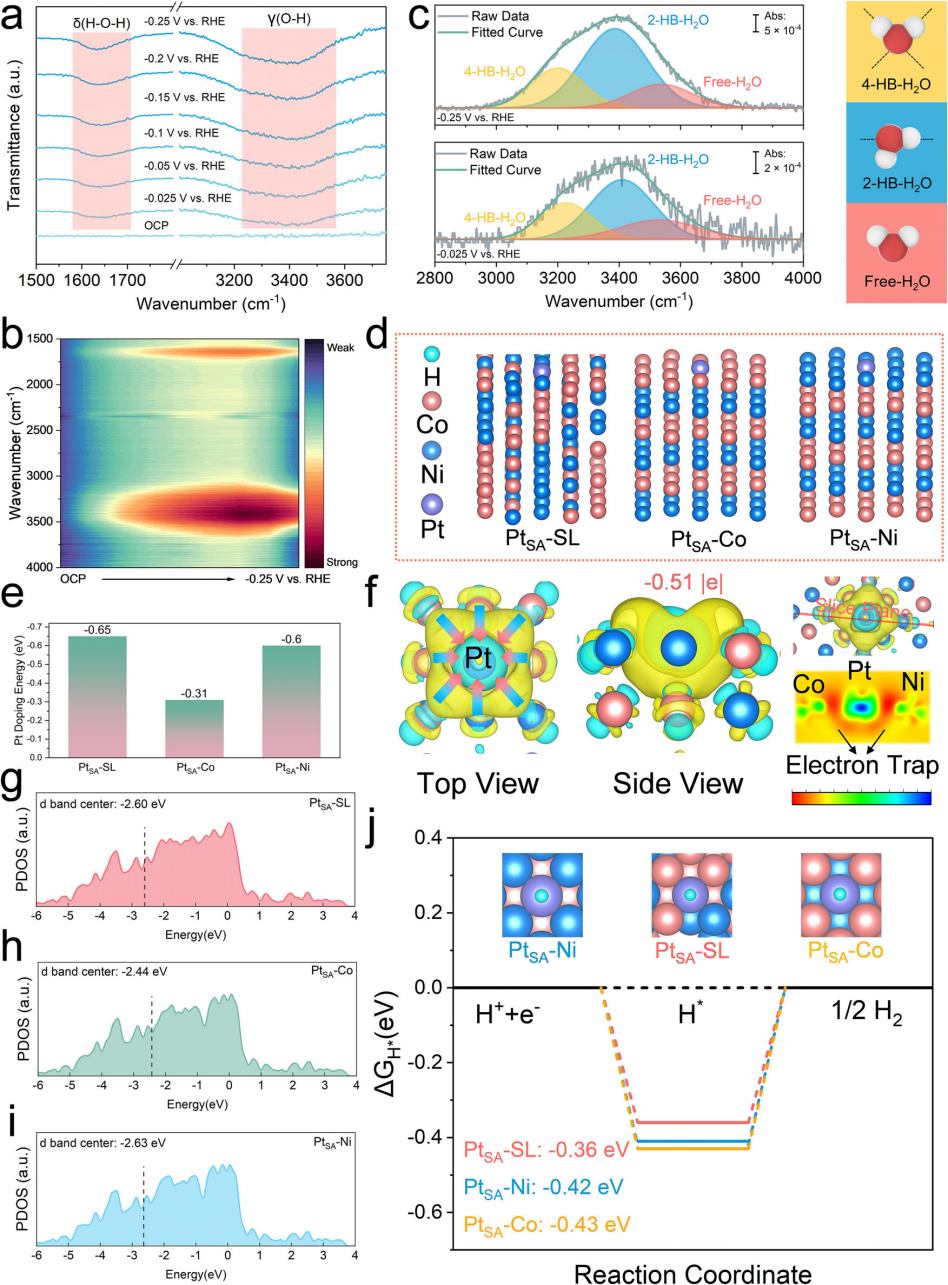
a) In situ ATR‐FTIR spectra of CoNiPt_SA_@G from OCP to −0.25 V versus RHE in 0.5 m H_2_SO_4_ electrolyte. b) Contour map of the in situ ATR‐FTIR spectra. c) deconvolution of the ν (O─H) peak in the in situ ATR‐SEIRAS spectra at −0.025 and −0.25 V versus RHE. d) Side views of the DFT calculation models for the optimized configurations of Pt_SA_─SL, Pt_SA_─Co, and Pt_SA_─Ni. e) Pt doping energy in the three configurations. f) Charge differential density map of Pt_SA_─SL configuration. The cyan part represents the depletion of electrons, and the yellow part represents the accumulation of electrons. The right‐hand side of the panel is the 2D intensity map and the respective slice plane. The isosurface value is set at 0.0022 e Bohr^−3^. g–i) PDOS and the corresponding *d* band center positions of Pt atoms for Pt_SA_─SL (g), Pt_SA_─Co (h), and Pt_SA_─Ni (i). j) Gibbs free energy diagrams of the H adsorption (Δ*G*_H*_) on the Pt site for e Pt_SA_─SL, Pt_SA_─Co, and Pt_SA_─Ni.

To investigate the catalytic mechanism of HER for the CoNiPt_SA_@G catalyst, in situ attenuated total reflectance Fourier transform infrared spectroscopy (ATR‐FTIR) was recorded within the potential range from open circuit potential (OCP) to −0.25 V versus reversible hydrogen electrode (RHE) in **Figure** **6**a. Two bands related to HER intermediates were detected when the potential was reduced from OCP to negative. The broad band located at 3420 cm^−1^ can be ascribed to the stretching vibrations of the O─H group in H_3_O^+^,^[^ ^59^ ^]^ whilst another one at ≈1600 cm^−1^ reflects the bending mode of water (δ (H─O─H)).^[^ ^60^, ^61^ ^]^ Figure 6b for the respective contour map shows that the IR signals are strongly dependent on the electrode potential, indicating that the signals are mostly excited from the interfacial water between electrolyte and catalyst surface. Since the intensity of δ (H─O─H) for the bending of water molecules is enhanced remarkably at the negative potential, it is inferred that the interfacial water may interact with the adsorbed hydrogen (H*) through the hydrogen bond of their hydrogen ends. The entire reaction pathway can thus follow the Heyrovsky step (H_3_O^+^ + e^−^ + H^*^ → H_2_ + H_2_O).^[^ ^60^ ^]^ Furthermore, we deconvoluted the O─H stretching vibration (ν (O─H)) band at −0.025 and −0.25 V versus RHE to investigate the interfacial water structure (Figure 6c). It can be deconvoluted into three sub‐bands located at ≈3228, 3391, and 3556 cm^−1^, which can be respectively assigned to 4‐coordinated hydrogen‐bonded water (4‐HB‐H_2_O), 2‐coordinated hydrogen‐bonded water (2‐HB‐H_2_O), and weak H‐bonded water (Free‐H_2_O).^[^ ^62^, ^63^ ^]^ The 2‐coordinated hydrogen‐bonded water is clearly the dominant species in the interface. The H‐bonded interfacial water can function as a proton acceptor to facilitate the proton transfer process. Additionally, the existence of free water is beneficial to reduce the local acidity of the CoNiPt_SA_@G catalyst surface, thus enhancing the stability of HER.^[^ ^64^, ^65^ ^]^ Additionally, in situ ATR‐FTIR spectra of CoNiPt_SA_@G from OCP to 1.6 V versus RHE in 1 m KOH electrolyte were recorded to probe the OER reaction mechanism, as shown in Figure S19 (Supporting Information). The band located at 1100–1108 cm^−1^ can be ascribed to the *OOH vibration,^[^ ^66^, ^67^ ^]^ while the band at ≈3300 cm^−1^ is assigned to the *OH species during OER.^[^ ^68^ ^]^ These results suggest that the reaction pathway for OER follows the adsorbate evolution mechanism path.^[^ ^69^, ^70^, ^71^ ^]^

Density functional theory (DFT) calculations were carried out to probe the interaction between Pt single‐atom sites and CoNi alloy and the origin of the remarkable catalytic activity. Herein, for the CoNi nanoalloy substrate, both disordered solid‐solution CoNi and ordered intermetallic CoNi configurations were considered.^[^ ^72^ ^]^ After rigorous tests of different initial configurations, three calculation models were built for the CoNiPt_SA_@G catalyst, including Pt SA in a solid solution of CoNi (Pt_SA_─SL), Pt SA in the Co layer of intermetallic CoNi (Pt_SA_─Co) and Pt SA in the Ni layer of intermetallic CoNi (Pt_SA_─Ni), as shown in Figure 6d and Figure S20 (Supporting Information). Figure 6e for the formation energy of Pt in the three configurations reflects that Pt single atoms are most thermodynamically stable when incorporated into the disordered CoNi solid solution (Pt_SA_─SL), with the least formation energy of −0.64 eV. More interestingly, the charge density difference analysis of the Pt_SA_─SL configuration in Figure 6f reveals a pronounced interfacial electron accumulation zone between the Pt center and the CoNi alloy substrate. Furthermore, the Bader charge analysis suggests that the Pt atom is −0.51 |e| close to the metallic state, demonstrating a pronounced electron transfer from CoNi to Pt. This result is consistent with XANES and XPS analysis. This interfacial electron‐rich area can be regarded as an electronic accumulation trap, enhancing the metal–support interaction. Such an electron trap effectively anchors the Pt atom and prevents its migration or aggregation. The electron‐rich state of Pt also contributes to catalytic performance by modulating the hydrogen adsorption.^[^ ^45^ ^]^

Figure 6g–i illustrates the respective projected density of states (PDOS) for Pt single atoms in the three different local environments, specifically Pt_SA_─SL, Pt_SA_─Co, and Pt_SA_─Ni configurations. Notably, the Pt *d*‐band center in the Pt_SA_─SL configuration is located at −2.60 eV, which lies between that of Pt_SA_─Co (−2.44 eV) and Pt_SA_─Ni (−2.63 eV). A moderate *d*‐band center position is conducive for balancing the adsorption and desorption properties of reaction intermediates on metal surfaces, thereby boosting the electrocatalytic performance of the catalyst.^[^ ^53^, ^73^, ^74^, ^75^ ^]^ To further evaluate the hydrogen adsorption properties, we calculated the Gibbs free energy of H adsorption (Δ*G*_H*_) on the Pt site for each configuration, as depicted in Figure 6j. Among them, the Pt_SA_─SL site exhibits a Δ*G*_H*_ value of −0.36 eV, which is closest to the thermoneutral value (0 eV) compared to Pt_SA_─Co (−0.42 eV) and Pt_SA_─Ni sites (−0.43 eV), indicating a more favorable thermodynamic profile for HER. These results suggest that Pt atoms embedded in a CoNi solid solution matrix (Pt_SA_─SL) provide the most active and well‐balanced adsorption sites for hydrogen evolution.

Finally, the values of Δ*G*_H*_ were computed for Co and Ni atoms in the Pt_SA_─SL configuration to assess their potential to compete with Pt single atoms. As shown in Figure S21 (Supporting Information), the Pt sites dominate the catalytic process, while Co and Ni serve primarily as electronic modulators. This is further corroborated by the charge density difference map (Figure S22, Supporting Information), which shows pronounced charge redistribution localized at the Pt–H interface, whereas Co and Ni atoms exhibit minimal perturbation, underscoring their stabilizing and supportive roles. For comparative purposes, two additional structural models were constructed, Pt─Co metal and Pt─Ni metal, respectively representing binary CoPt@G and NiPt@G metallic systems (Figure S23, Supporting Information). In both cases, the Δ*G*_H*_ values for hydrogen adsorption are less optimal (Figure S24, Supporting Information), being −0.40 eV for Pt─Co and −0.47 eV for Pt─Ni. These findings further highlight the superior hydrogen adsorption capability and electronic synergy of the Pt_SA_─SL sites which are firmly embedded within the CoNi solid solution framework.

## 5 Conclusion

In this study, we have demonstrated a novel and effective strategy for the synthesis of Pt SACs with a large mass loading of 3.82 wt.% and a high Pt areal density of 10.6 atoms nm^−2^ using a one‐step, DCAD approach. This method is remarkable for its simplicity, eliminating the need for additional chemical reagents; cost‐effectiveness; rapid synthesis (in 30 mins); and scalability, achieving gram‐scale production—significantly surpassing the milligram‐scale outputs reported in previous studies. The approach also proved efficient and versatile, producing a superior HER electrocatalyst which outperforms many reported catalysts in having a low overpotential (23 mV at 10 mA cm^−2^) and a high stability during a 120 h chronopotentiometry test. Looking ahead, we aim to expand the application of this strategy by developing a synthesis library for other Pt‐group metals such as Pd and Ru, further demonstrating the universality of the method. Additionally, efforts will be directed toward scaling up production to the kilogram and even ton scale for industrial‐level demonstrations. This goal appears achievable given that arc discharge systems are already commercially deployed for the bulk production of materials such as fullerenes and graphene.^[^ ^76^ ^]^ Furthermore, powering the DCAD system with renewable energy sources could render the process not only scalable but also sustainable, offering a green and practical pathway for the mass production of Pt‐group metal SACs for clean energy applications.

## Conflict of Interest

The authors declare no conflict of interest.

## Supporting information



Supporting Information

## Data Availability

The data that support the findings of this study are available in the supplementary material of this article.
