# Supplementary material for: Facile Synthesis of High Areal Density and Stable Pt Single‐Atom Electrocatalysts by Arc Discharge Atomization and CoNi Trapping
Source: Adv Sci (Weinh). 2025 Aug 27;12(42):e11806. doi: 10.1002/advs.202511806 (PMC12622532; doi:10.1002/advs.202511806)
Supplement: Supplementary file 1 — Supporting Information [file ADVS-12-e11806-s001.docx]

Supporting Information

**Facile Synthesis of High Areal Density and Stable Pt Single-Atom Electrocatalysts by Arc Discharge Atomization and CoNi Trapping**

*Hongzhe He, Xiaoqiong Ren, Ruoqun Zhang, Sasha Yang, Jinxing Gu, Ke Wang, Binbin Qian, Ning Chen*, Lian Zhang*, Jianglong Yu, Yuan Cheng, Baiqian Dai**

1. **Experimental section**
   1. **Chemicals**

Cobalt powder (99.9 % metals basis) and Nickel powder (99.99%, metals basis) were purchased from Shanghai Macklin Biochemical Technology Co., Ltd. Platinum dioxide (PtO_2_, 98%) was purchased from Adamas-beta. N, N-Dimethylformamide (DMF, >99.9% (GC)) was purchased from Shanghai Macklin Biochemical Technology Co., Ltd. Graphite powder and Graphite rod for direct current arc discharge (DCAD) was purchased from Foshan Yinyihui Graphite Mould Factory. The 20 wt% Pt/C catalyst was purchased from Beijing Innochem Technology Co., Ltd. 0.5 M H_2_SO_4_ and 1 M KOH electrolyte were purchased from Codow Chemicals, Guangzhou Howei Pharma Technology Co., Ltd. Dupont Nafion D520 (5 wt%) was purchased from Suzhou Sinero Technology Co., Ltd.

- 1. **Synthesis of CoNiPt_SA_@G and Reference Catalysts**

**Synthesis of CoNiPt_SA_@G:** 11 mmol Co, 11 mmol Ni and 1 mmol PtO_2_ with 54 mmol Graphite powder were mixed together. The mixed powder was then filled into a customized hollow graphite rod (6 mm ID × 115 mm length). The graphite rod was annealed in a tubular furnace under 1000 ℃ at N_2_ atmosphere for 10 h (heating rate: 10 ℃ min ^-1^). The annealed rod was then assembled as anode in the DCAD chamber. An initial distance of approximately 10 mm was set between the anode and cathode. The chamber was first evacuated and then purged with 200 Torr Helium. DCAD was initiated at 100 A, with continuous cooling water flowing through the outer wall. Upon current application, a stable arc formed, generating a high-temperature plasma core (~6000 K), leading to rapid anode evaporation. The process was terminated once the graphite anode was fully consumed, typically within 30 minutes. The carbon soot deposited on the cooling water jacketed chamber wall was collected and denoted as CoNiPt_SA_@G.

**Synthesis of CoNiIr_SA_@G:** CoNiIr_SA_@G was synthesized follow the same procedure whilst the 1 mmol PtO_2_ precursor were replaced by 1 mmol IrO_2_.

**Synthesis of Other Reference Catalysts:** The reference catalysts were also prepared per the aforementioned procedure with the absence of one or even two metal precursors. For CoPt@G, the Ni metal precursor was ruled out, and the quantity of Co was doubled to 22 mmol Co which is further blended with 1mmol PtO_2_ blended into 54 mmol Graphite powder. For NiPt@G, the Co metal precursor was dropped out; and for Pt@G sample, 1mmol PtO_2_ and 108 mmol Graphite powder were mixed together with the absence of both metal precursors. Likewise, the CoNi@G sample was prepared with 11 mmol Co, 11 mmol Ni and 54 mmol Graphite powder.

**Thermal Treatment of CoNiPt_SA_@G**: 200 mg CoNiPt_SA_@G catalyst was placed in a corundum crucible and thermally treated in a tubular furnace under a N_2_ atmosphere at 1000 ℃ for 2 h with a controlled heating rate of 5 ℃ min^-1^ after which the resulting material was designated as CoNiPt_SA_@G-1000.

- 1. **Material Characterizations**

**Benchtop Characterizations:** Scanning electron microscopy (SEM) was performed on a ZEISS Sigma 300. Transmission electron microscope (TEM) and scanning transmission electron microscopy-energy dispersive X-ray spectroscopy (STEM-EDX) were performed on a Tecnai G2 F20 field-emission microscope operated at an accelerating voltage of 200 kV. Atomic resolution TEM images were obtained from a 300 kV FEI Themis Z equipped with double spherical aberration correctors. X-ray photoelectron spectroscopy (XPS) measurements were carried out on a Thermo Scientific K-Alpha spectrometer equipped with an Al anode (Al Kα = 1486.6 eV). XPS spectra fitting was carried out on the Avantage software with C calibration. Raman spectra were obtained on a Horiba LabRAM HR Evolution using a 532 nm wavelength laser. Inductively coupled plasma-optical emission spectroscopy (ICP-OES) results for spent leachates were obtained from Agilent 5110. N_2_ adsorption and Brunauer-Emmett-Teller (BET) surface area analysis was conducted on Micromeritics 3Flex.

**Synchrotron Characterizations:** Synchrotron Powder Diffraction (PD) test was carried out on the Powder Diffraction beamline at the Australian Synchrotron ANSTO with a refined wavelength of 0.59031 Å. X-ray absorption spectroscopy (XAS) spectra was tested on Australian Synchrotron ANSTO (beamline MEX-1). X-ray absorption near edge structure (XANES) and extended X-ray absorption fine structure (EXAFS) data were processed through the Athena program implemented in Demeter software.^[1]^ The wavelet transform of EXAFS spectra was performed via a hamaFortran software^[2]^.

- 1. **Electrochemical Measurements**

All the electrochemical measurements were conducted by an electrochemical workstation (CHI 760E). Hydrogen evolution reaction (HER) and oxygen evolution reaction (OER) performance tests were conducted via a three-electrode system.

**Working Electrode Preparation:** For catalyst ink preparation, 20 mg catalyst powder was firstly dispersed in 1970 μL DMF and 30 μL 5% Nafion, which was subjected to ultrasonication for 1 h to achieve a uniform dispersion of the catalyst ink. Subsequently, 80 μL catalyst ink was drop-cast on both sides of a rectangular carbon cloth with a working area of 0.5 cm^2^ (0.5 cm in width). The ink-coated carbon cloth was clamped by a glassy-carbon electrode holder as the working electrode. The total mass load of catalysts was 0.8 mg.

**HER Activity Tests:** HER activity tests were conducted in N_2_-saturated 0.5 M H_2_SO_4_ electrolyte. For the three-electrode configuration, the ink-coated carbon cloth was set as the working electrode. The reference electrode and counter electrode were Ag/AgCl (saturated KCl) electrode and a carbon rod with a diameter of 6 mm, respectively. The calibration of Ag/AgCl reference electrode with respect to the reversible hydrogen electrode (RHE) was conducted via the equation: E_RHE_= E_Ag/AgCl_ + 0.197+ 0.059 × pH. Polarization curve was obtained by linear sweep voltammetry (LSV) with a scanning rate of 5 mV s^-1^ with 90% iR compensation. Cyclic voltammetry (CV) tests were carried out in a non-faradaic potential range of -0.17 V versus Ag/AgCl to -0.27 V versus Ag/AgCl at the scanning rates of 20, 40, 60, 80, 100 and 120 mV s^-1^ to obtain the specific double-layer capacitance (C_dl_) data by plotting the current difference (Δ_j_) against the scanning rate. By fitting the linear part of the Tafel plots to the Tafel equation η = a + b log j (a is the intercept, η is the overpotential, b is the Tafel slope and j is the current density), Tafel slopes were obtained. Exchange current density (j_0_) was estimated by extrapolating the linear region of Tafel plots back to zero overpotential to indicate the kinetics of electrocatalysts. Electrochemical impedance spectroscopy (EIS) measurement was carried out at an overpotential of 53 mV with a frequency ranging from 0.1 to 10^5^ Hz and an amplitude of 5 mV. An accelerating durability measurement was evaluated using 7,000 continuous cycles from 0.2 to -0.3 V versus RHE with a scanning rate of 200 mV s^-1^. The LSV before and after CV cycles were recorded with a scanning rate of 5 mV s^-1^ and 90% iR compensation. Chronopotentiometry (CP) was tested at a current density of 10 mA cm^-2^.

**OER Activity Tests:** OER activity tests were conducted in O_2_-saturated 1 M KOH electrolyte. A Hg/HgO electrode (1 M KOH) was used as the reference electrode for the OER tests. The calibration of Hg/HgO reference electrode with respect to the RHE was conducted via the equation: E_RHE_= E_Hg/HgO_ + 0.098 + 0.059 × pH. Polarization curve was obtained by linear sweep voltammetry (LSV) at a scanning rate of 5 mV s^-1^ with 90% iR compensation. Tafel slopes were obtained following the same method in HER test. EIS was performed at an overpotential of 340 mV and the frequency ranging from 0.1 to 10^5^ Hz and an amplitude of 5 mV.

**Overall Water Splitting Performance Tests:** Overall Water Splitting Tests were conducted via a two-electrode system. In 0.5 M H_2_SO_4_ electrolyte, IrO_2_ (+) || 20 wt% Pt/C (-) and IrO_2_ (+) || CoNiPt_SA_@G (-) configurations were tested. In 1 M KOH electrolyte, CoNiPt_SA_@G (+) || 20 wt% Pt/C (-) and IrO_2_ (+) || 20 wt% Pt/C (-) assemblies were tested. The LSV curves were obtained at the voltage range of 1.1-2.0 V with a scan rate of 5 mV s^-1^.

**Turnover Frequency (TOF) Calculation:** TOF values were calculated via the following equation:

$$TOF= \frac{j\times N_{A}}{n\times F\times\tau}$$

where j, N_A_, n, F, and τ are current density Avogadro number (6.023 × 10^23^ mol^-1^), number of electrons transferred (2 for HER and 4 for OER), Faraday constant (96485 C mol^-1^), and the number of active sites, respectively. For the determination of τ in CoNiPt_SA_@G, we assume all the Pt atoms are active and accessible to the electrolyte and thus the maximum number of active sites can be calculated according to the Pt content characterized by ICP-OES.^[3,4]^ Likewise, τ in 20 wt% Pt/C and RuO_2_ were calculated on the assumption that all Pt or Ru atoms acted as active sites.

**Electrochemical Surface Area (ECSA) Calculation:** The ECSA of an electrode was determined by the following equation:

$$ECSA=\frac{C_{\mathrm{dl}}}{C_{s}}$$

where C_dl_ refers to the electrochemical double-layer capacitance (Cdl) obtained from CV test. C_s_ is the specific capacitance for a flat surface, which is generally found to be in the range of 20-60 μF cm^-2^. Herein, a C_s_ value of 35 μF cm^-2^ was used for HER ECSA calculation^[5,6]^.

**In Situ Attenuated Total Reflectance Fourier transform infrared spectroscopy (ATR-FTIR) Measurements:** In Situ ATR-FTIR spectra was recorded on a PerkinElmer Spectrum 3 with a customized sample cell (Hefei In-situ Technology Co., LTD.). A silicon crystal was used as the infrared crystal. For in situ HER testing, the experiment is conducted in a 0.5 M H_2_SO_4_ electrolyte solution, which is first purged with high-purity nitrogen (N_2_) for 30 minutes. The electrochemical setup consists of a catalyst-coated glassy carbon (GC) electrode, an Ag/AgCl reference electrode, and a graphite counter electrode. The infrared background was removed before measurement. The spectra were collected under applied potentials at voltages from the open circuit potential (OCP) to -0.25 V versus RHE. For in situ OER testing, the test was performed in a 1 M KOH electrolyte solution, with oxygen gas purging for 30 minutes. Spectra were recorded at various applied potentials from OCP to 1.6 V versus RHE using an Hg/HgO reference electrode and a graphite counter electrode.

- 1. **Theoretical Calculations**

The density functional theory (DFT) calculations were carried out by using the Vienna ab initio simulation package (VASP). All geometry optimization and energy calculations were performed using the projector-augmented wave (PAW) method^[7]^. The generalized gradient approximation (GGA) functional of Perdew, Burke and Enzerhof (PBE) was used to analyze the exchange and correlation potential^[8]^. In addition, the Grimme method for DFT-D3 correction was selected for Van der Waals interactions. The surface models of CoNi (200), Co (200) and Ni (200) were constructed by 3 × 2 supercells with a thickness of six atomic layers. The solid-solution CoNi model was constructed by randomly substituting lattice sites in an FCC framework with Co and Ni atoms in a 1:1 ratio. The bottom atoms of the optimized Pt_SA_-SL, Pt_SA_-Co, Pt_SA_-Ni and Pt-Co metal and Pt-Ni metal were fixed during the calculations, while all the adsorbed hydrogen atoms were relaxed. The k-point samplings of the Brillouin zone were set at 3 x 3 x 1, the vacuum layer was set at 20 Å, and the energy cut-off of 600 eV. Geometrical optimization of the system was carried out until both the energy and atomic forces converged to 1E-5 eV and 0.01 eV/Å. The reaction Gibbs free energy(eV) was calculated by: ∆G = ∆E + ∆ZPE - T∆S ^[9]^**,** where ∆E, ∆ZPE and ∆S were the change of total energy, zero-point energy, and entropy, respectively.

Note that we did not consider the (111) surface of the CoNi alloy although this surface has been reported as the active surface for a variety of reactions (HER, OER, CO oxidation, etc.)^[10–12]^. However, it is not the case in this study. We disregarded it during the calculation due to lack of model stability.

1. **Supplementary Figures**


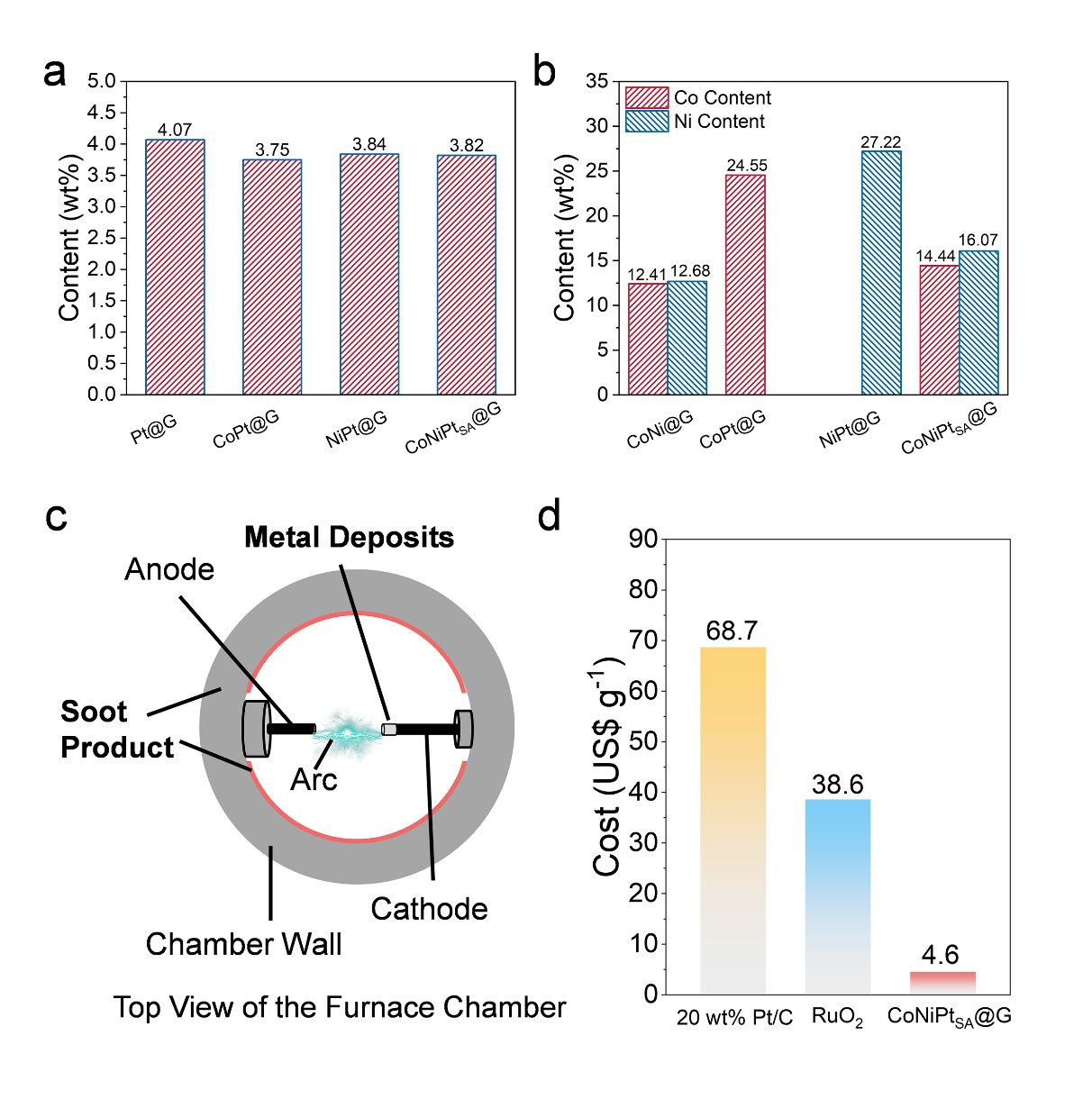


**Figure S1 Composition Quantification and yield demonstration. a**, ICP-OES results of Pt content. **b**, ICP-OES results of Co & Ni content. **c**, The schematic diagram of DCAD furnace chamber. **d**, Materials cost comparison of the CoNiPt_SA_@G catalyst and commercial catalysts**.**

**
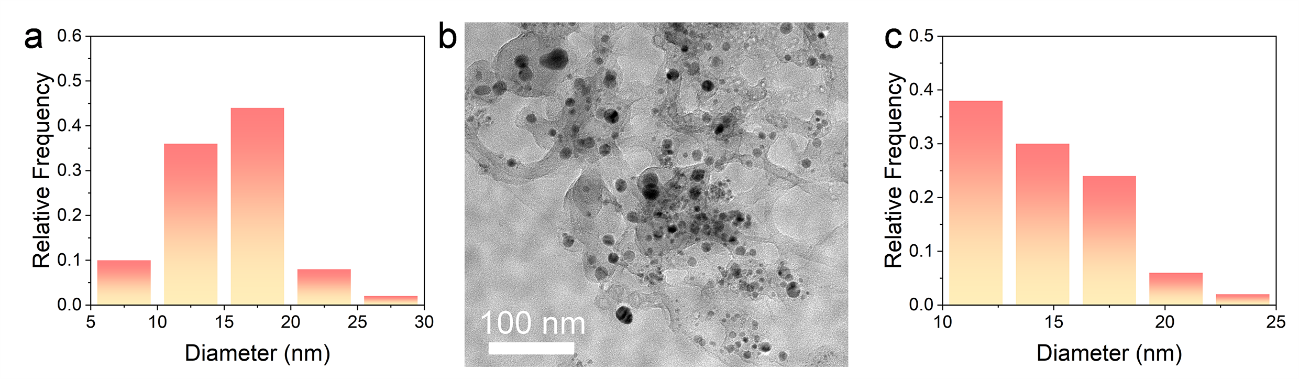
**

**Figure S2 Morphology Analysis of CoNiPt_SA_@G. a**, Size distribution of fibrous structures in CoNiPt_SA_@G. **b**, TEM images of CoNiPt_SA_@G for measurement of particle diameters. **c**, Size distribution of particles in CoNiPt_SA_@G.

**
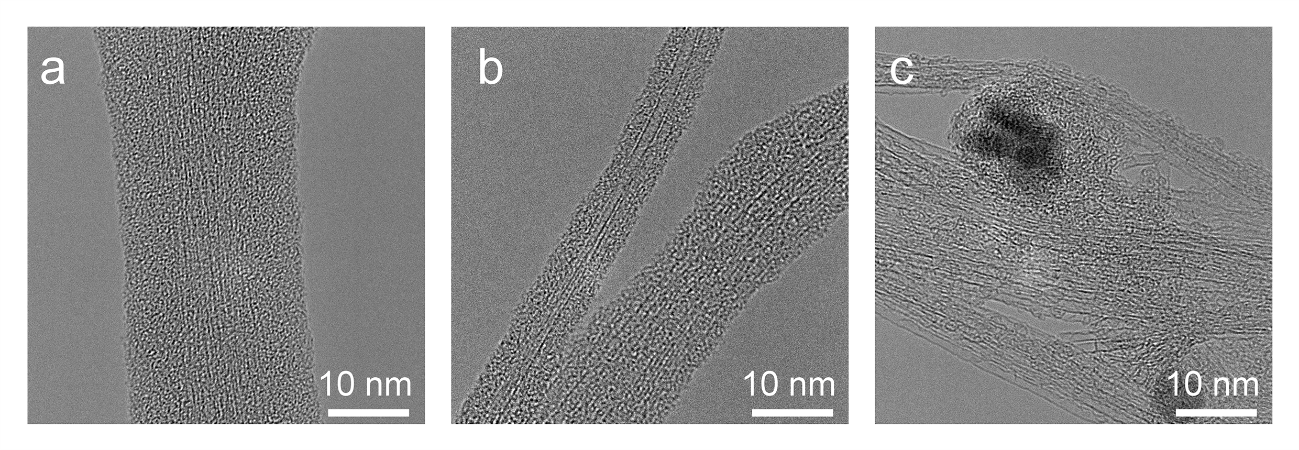
**

**Figure S3 Identification of Carbon Nanotubes. a-c,** TEM images of Carbon Nanotube Structure in CoNiPt_SA_@G.

**
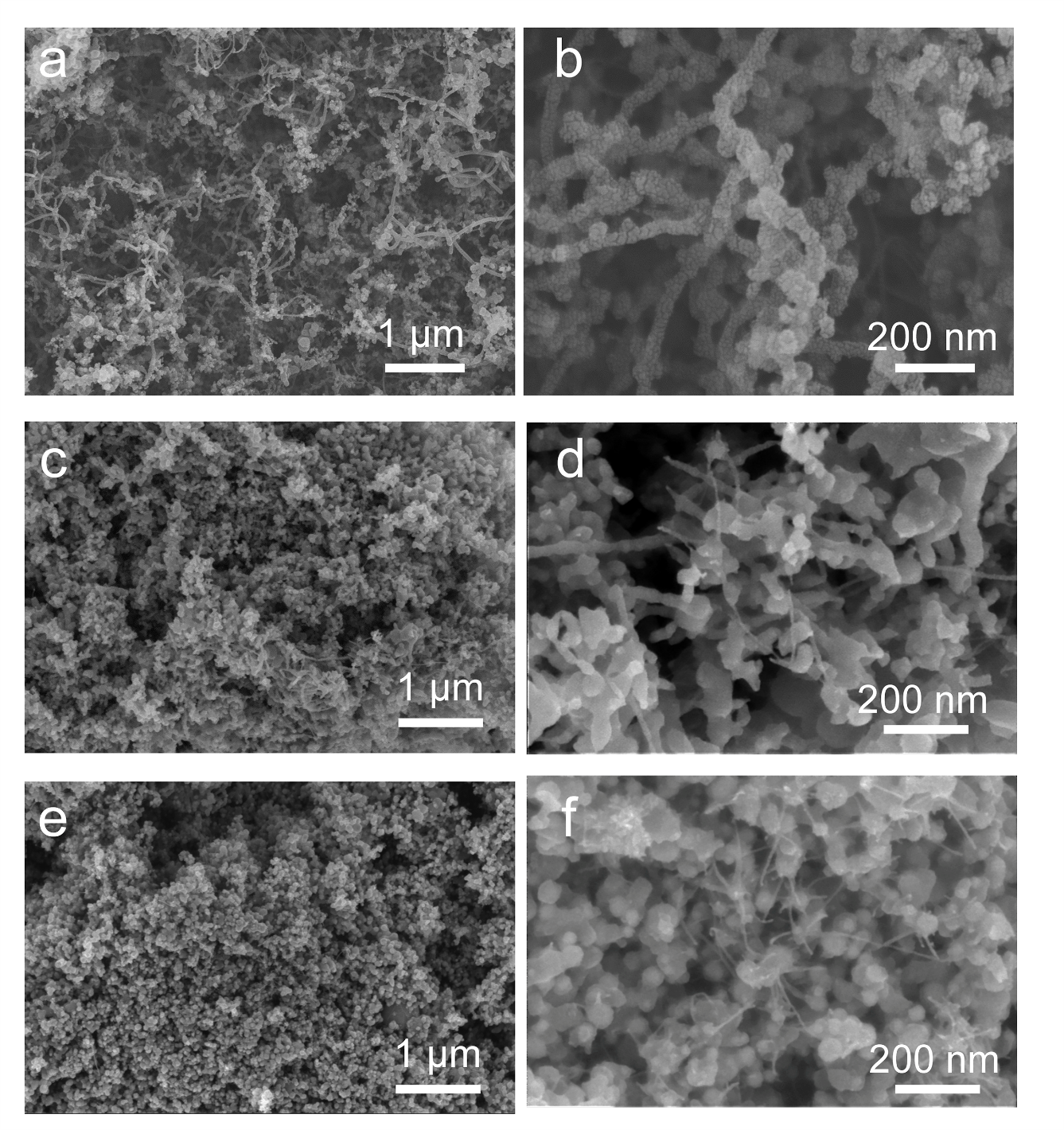
**

**Figure S4 Morphology Characterizations. a**, **b**, SEM images of CoNi@G. **c**, **d** SEM images of CoPt@G. **e**, **f**, SEM images of NiPt@G.

**
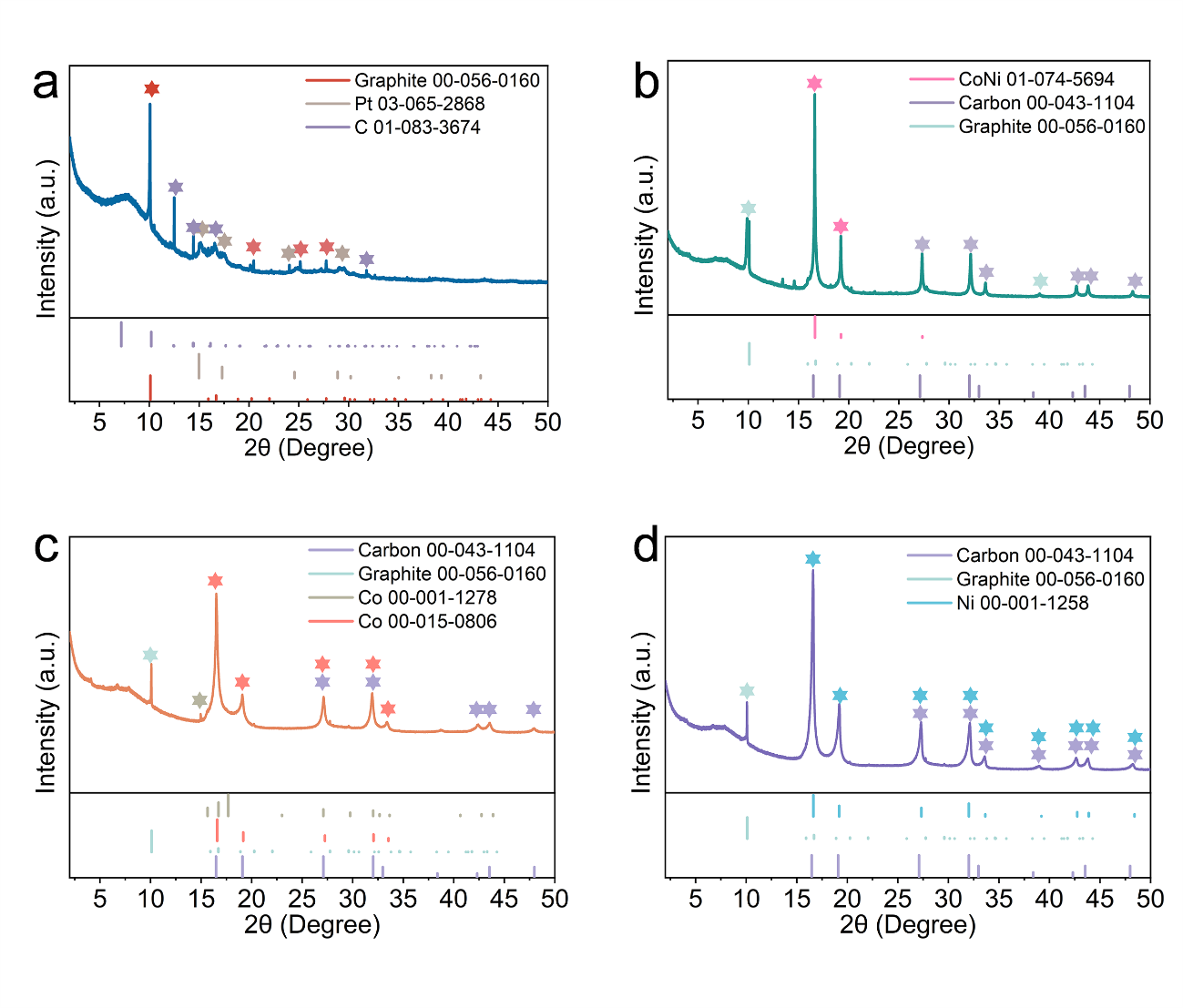
**

**Figure S5 Structural Analysis. a,** Powder diffraction (PD) pattern of Pt@G. **b** PD pattern of CoNi@G. **c,** PD pattern of CoPt@G. **d,** PD pattern of NiPt@G. The patterns were collected at the Australian Synchrotron ANSTO with a refined wavelength of 0.59031 Å.

**
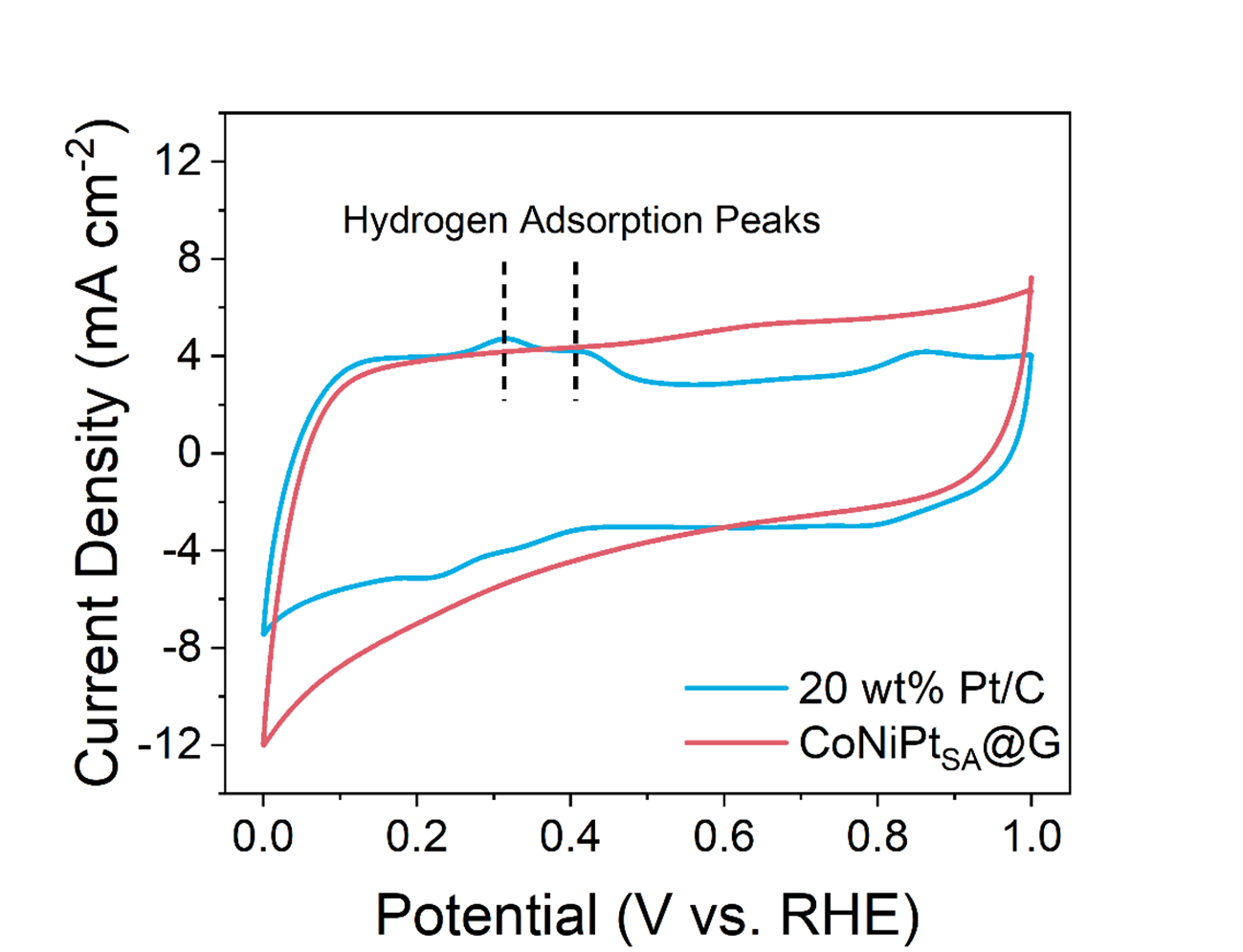
**

**Figure S6** Underpotentially deposited hydrogen of CoNiPt_SA_@G and 20 wt% Pt/C. Cyclic voltammetry (CV) measurements curves collected at a sweep rate of 50 mV s^-1^ in the potential range of 0 to 1.0 V versus RHE in 1 M KOH.

At around 0.3 V and 0.4 V versus RHE, the hydrogen adsorption/desorption characteristic peaks had appeared for 20 wt.% Pt/C, which can be ascribed by the existence of Pt nanoparticles. Particularly, the CV curve of CoNiPt_SA_@G exhibited no hydrogen adsorption/desorption characteristic peaks, indicating that Pt atoms in CoNiPt_SA_@G were mainly atomically dispersed.

**
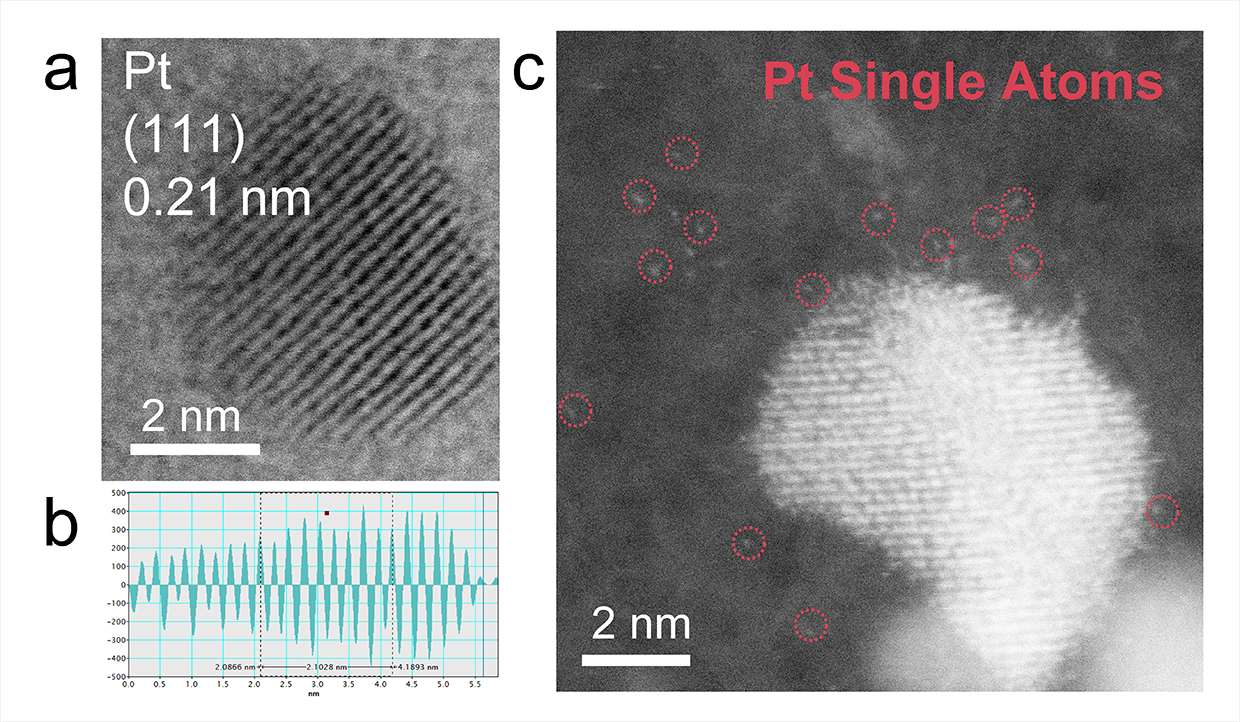
**

**Figure S7 Visualization of the structure of Pt@G. a**, **b**, HR-TEM images of Pt@G (a) and the corresponding lattice distance (b). **c**, AC-HAADF-STEM image of Pt@G. Marked in red circles are Pt single atoms.


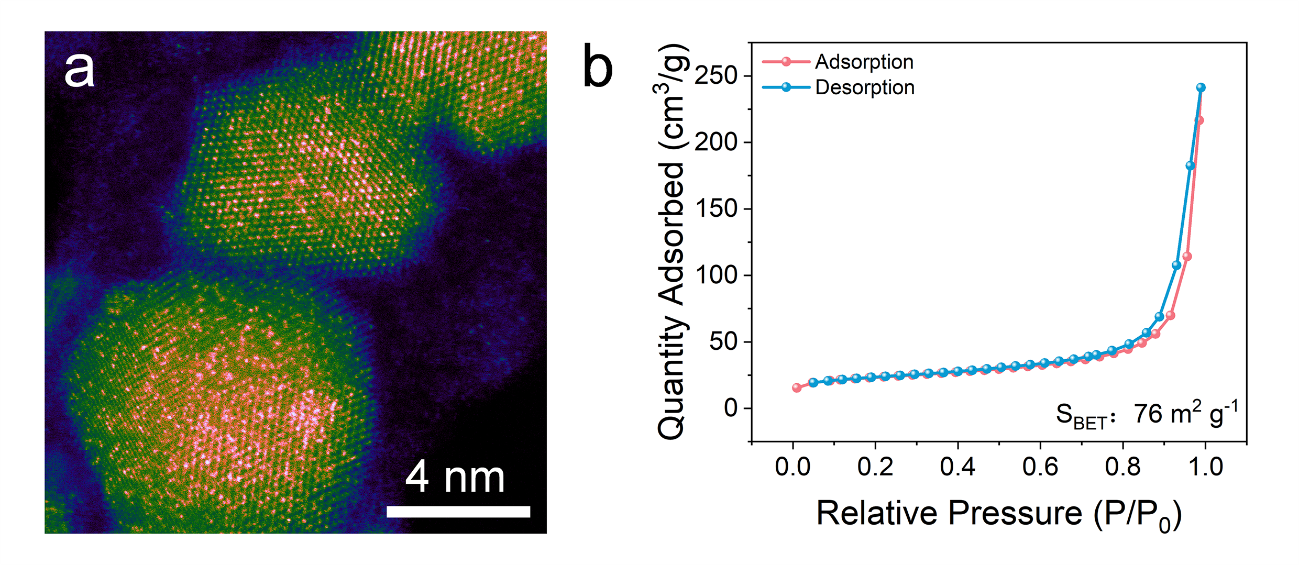


**Figure S8 Confirmation of high areal density of Pt atoms. a**, HR-HAADF-STEM image of CoNiPt_SA_@G. **b**, N_2_ adsorption/desorption isotherms of CoNiPt_SA_@G.

**
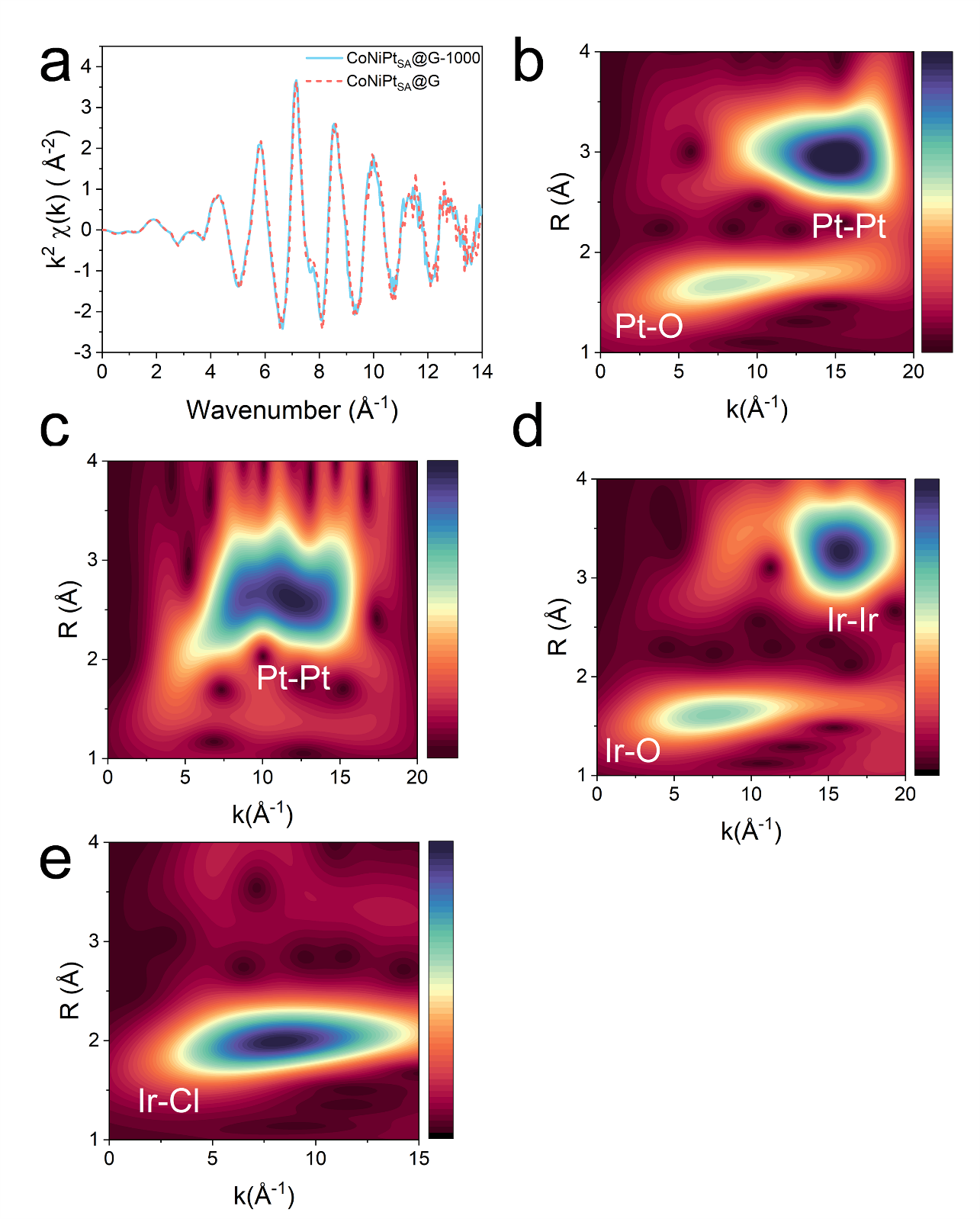
**

**Figure S9 EXAFS analysis of synthesized catalysts and standards.** **a,** k^2^-weighted Pt L_3_-edge EXAFS oscillations in k space of CoNiPt_SA_@G and CoNiPt_SA_@G-1000. **b,** WT maps for the k^3^-weighted EXAFS signal of PtO_2_. **c,** WT maps for the k^3^-weighted EXAFS signal of Pt@G. **d,** WT maps for the k^3^-weighted EXAFS signal of IrO_2._ **e,** WT maps for the k^3^-weighted EXAFS signal of IrCl_3_.

The WT map of PtO_2_ (**Figure S9b**) shows the maximum intensity near R ≈ 2.9 Å and k ≈ 14.5 Å^-1^, which corresponds to the Pt-Pt coordination. Except for the signal of Pt-Pt coordination, Pt-O coordination signal can be observed at R ≈ 1.6 Å and k ≈ 6.1 Å^-1^. Similarly, the WT of IrO_2_ (**Figure S9d**) exhibits two coordination signals. One is Ir-O (R ≈ 1.5 Å and k ≈ 7.5 Å^-1^) coordination and the other is Ir-Ir Coordination (R ≈ 3.4 Å and k ≈ 16.0 Å^-1^). **Figure S9e** for the the WT of IrCl_3_ shows the Pt-Cl coordination (R ≈ 2.0 Å and k ≈ 7.7 Å^-1^).

**
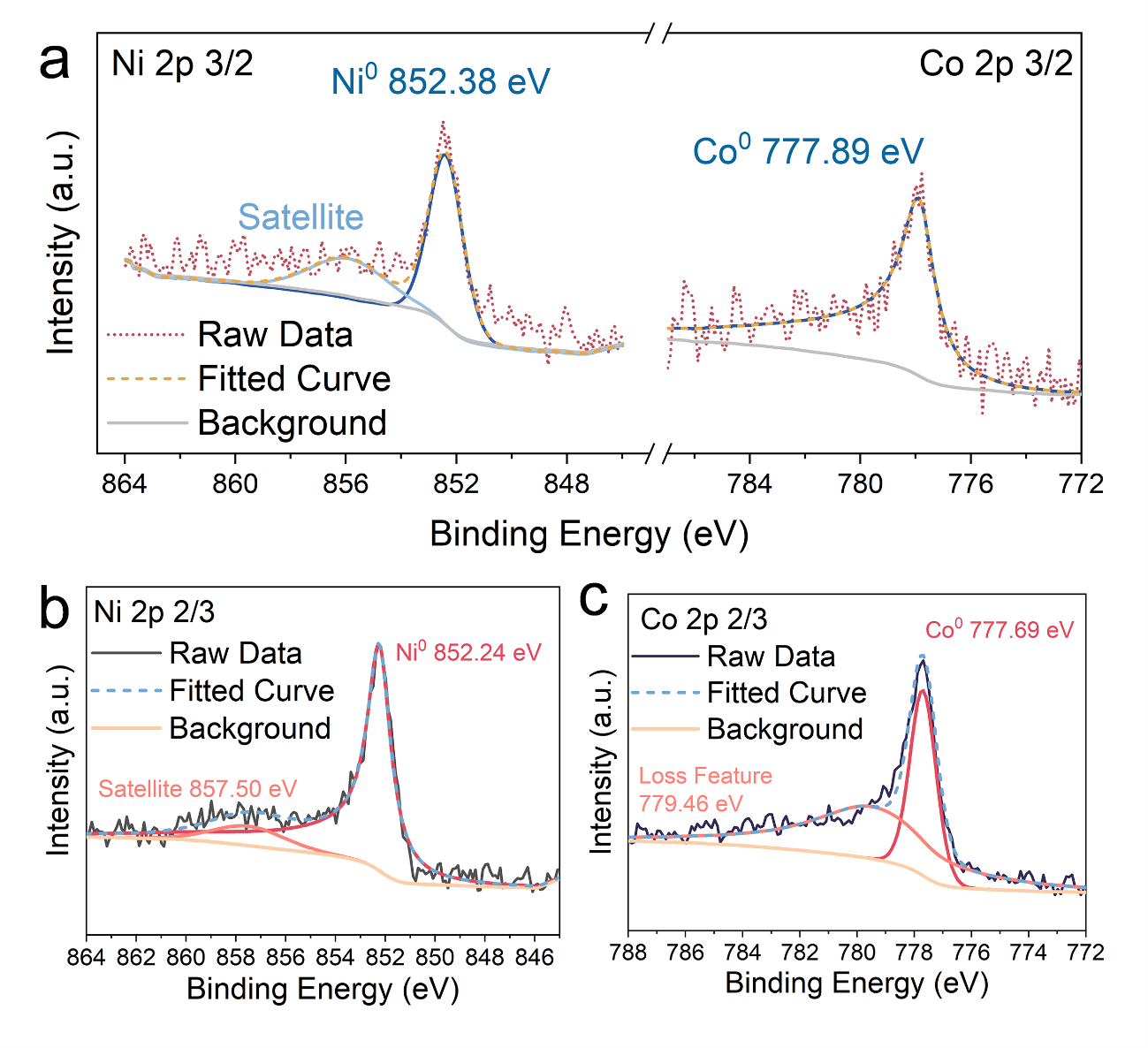
**

**Figure S10 XPS Spectra of Co and Ni. a,** Co 2p 3/2 and Ni 2p 3/2 spectra of CoNi@G. **b**, Ni 2p 3/2 of spectrum NiPt@G. **c**, Co 2p 3/2 of spectrum CoPt@G.

**
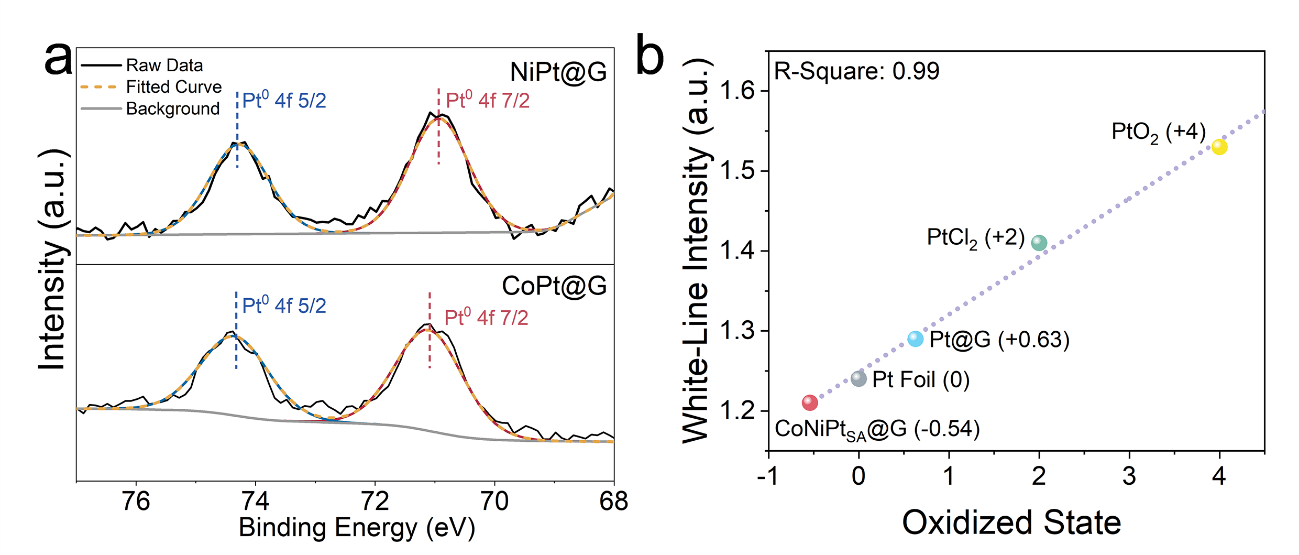
**

**Figure S11 Electronic structure analysis. a,** XPS Pt 4f Spectra of NiPt@G and CoPt@G**. b,** Fitted curve correlating the average oxidation state of Pt in the CoNiPt_SA_@G, PtCl_2_, Pt foil, and PtO_2_ at Pt L_3_ edge.

The presence of a small amount of Pt^2+^ in the Pt 4f Spectra of Pt@G (**Figure 4b**) is attributed to the coordination of Pt single atoms with C atom, causing partial electron migration from Pt to C^[13,14]^. This is supported by the observation of Pt single atoms in Pt@G in TEM image (Figure S7).

**
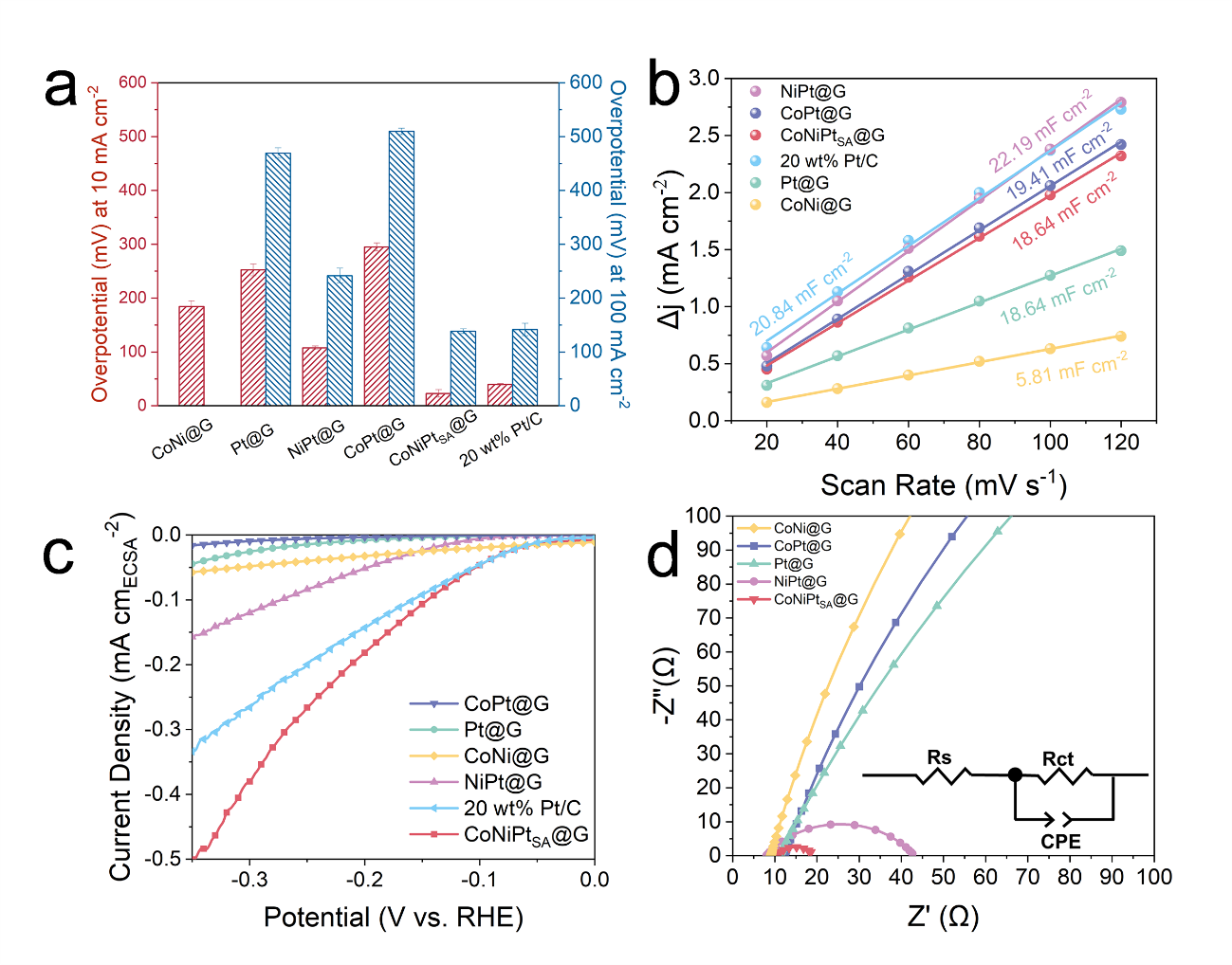
**

**Figure S12 HER Activity Evaluation. a**, Overpotential values at 10 mA cm^-2^ and 100 mA cm^-2^. **b**, Capacitive Δ j = j_a_ - j_c_ as a function of scanning rate in 0.5 M H_2_SO_4_. **c**, Polarization curves normalized by the ECSA. **d**, Nyquist plots at an overpotential of 0.05 V versus RHE (Insert: The equivalent circuit for the fitting of R_ct_ values).

**
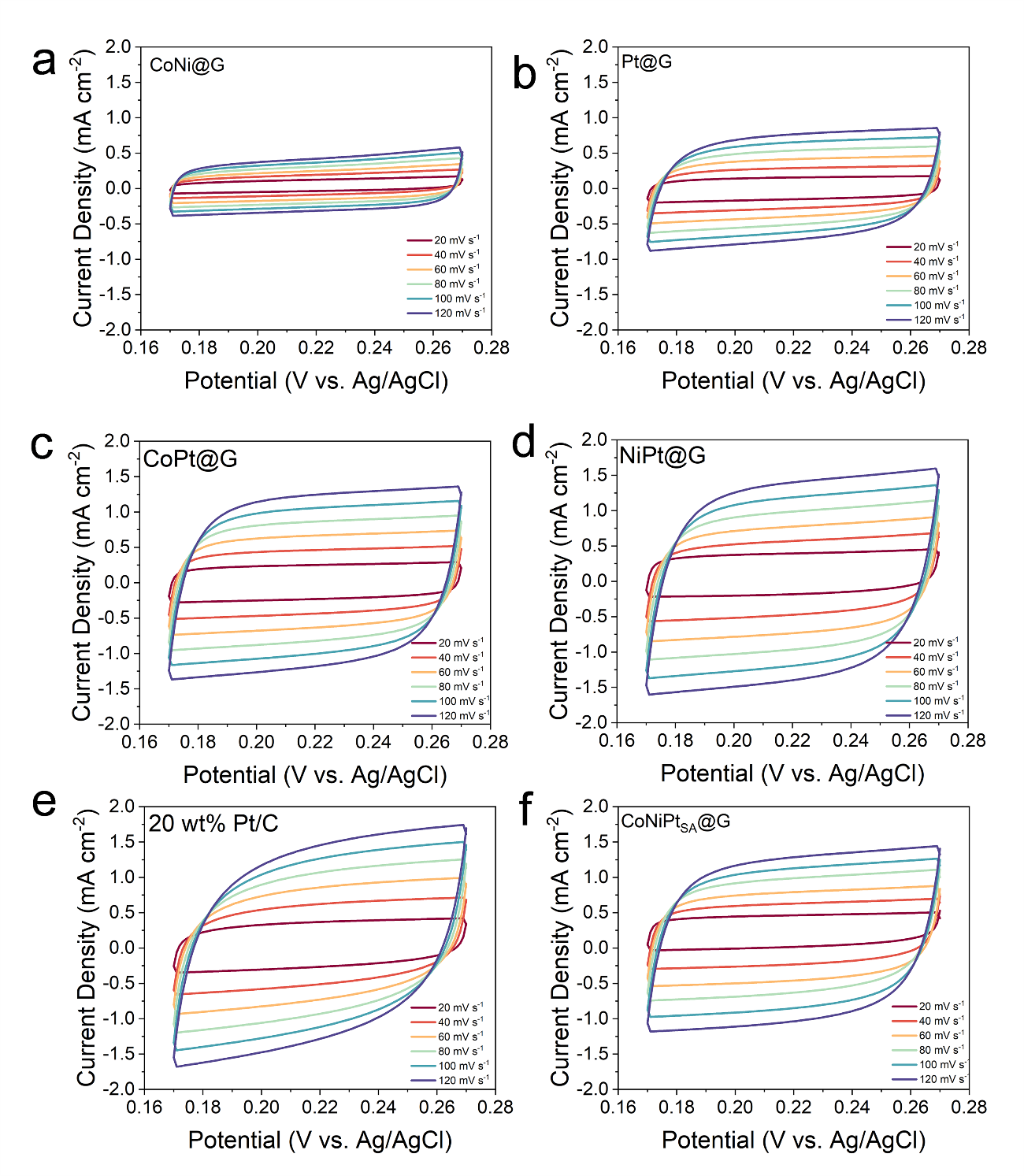
**

**Figure S13** CV Curves Measured in a Non-Faradaic Region at Different Scanning Rates.

**
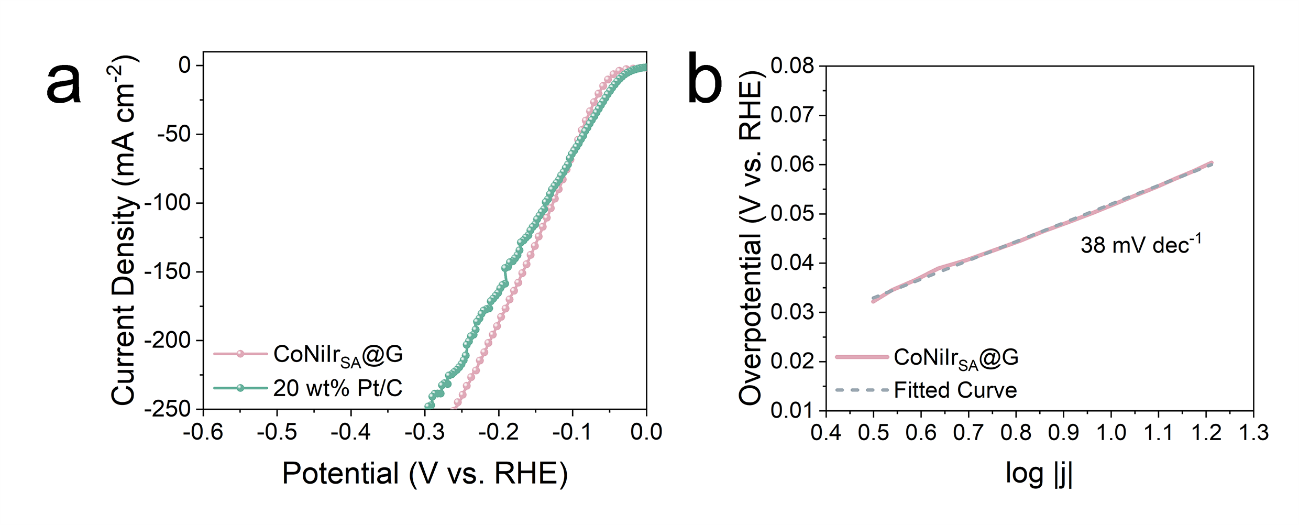
**

**Figure S14 HER Activity Evaluation. a,** Polarization curves of CoNiIr_SA_@G and commercial 20 wt.% Pt/C catalyst in the 0.5 M H_2_SO_4_ electrolyte after 90% i-R compensation**. b,** Tafel slope of CoNiIr_SA_@G.


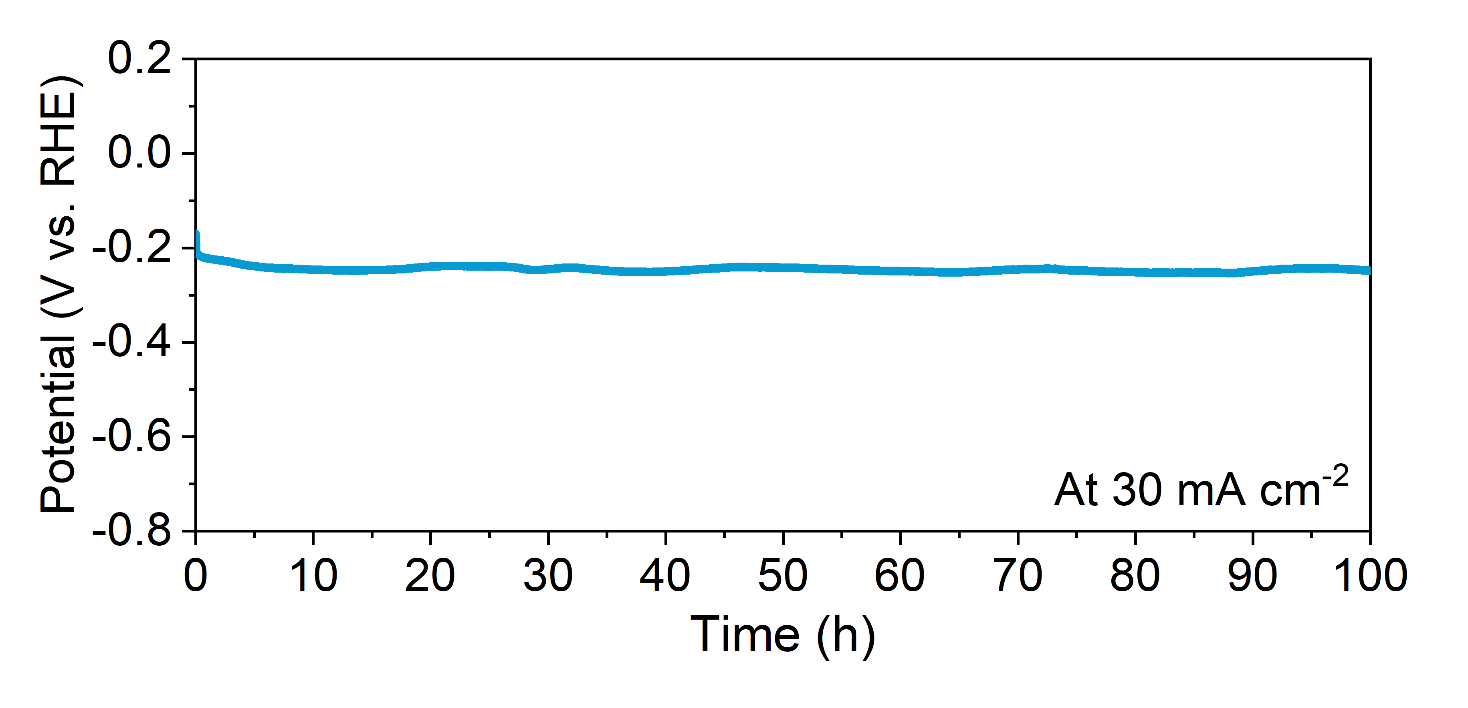


**Figure S15** CP test of CoNiPt_SA_@G at a current density of 30 mA cm^-2^.

**
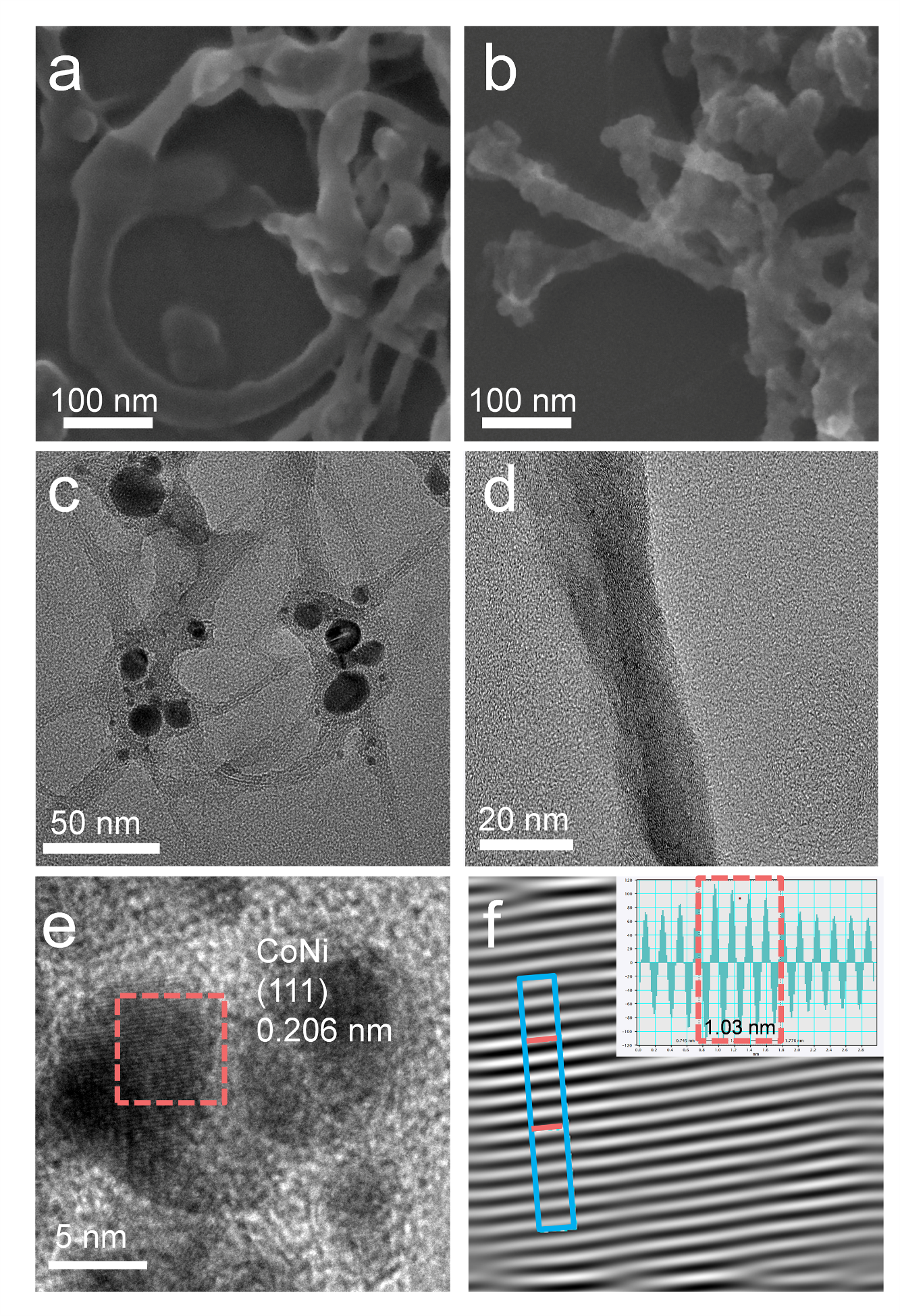
**

**Figure S16 Morphology and Structure Characterizations of CoNiPt_SA_@G after HER Stability Test. a-b**, SEM Images of Spent CoNiPt_SA_@G. **c-d**, TEM Images of Spent CoNiPt_SA_@G. **e**, HR-TEM image of Spent CoNiPt_SA_@G. **f**, The lattice space of the corresponding area in **e**.

**
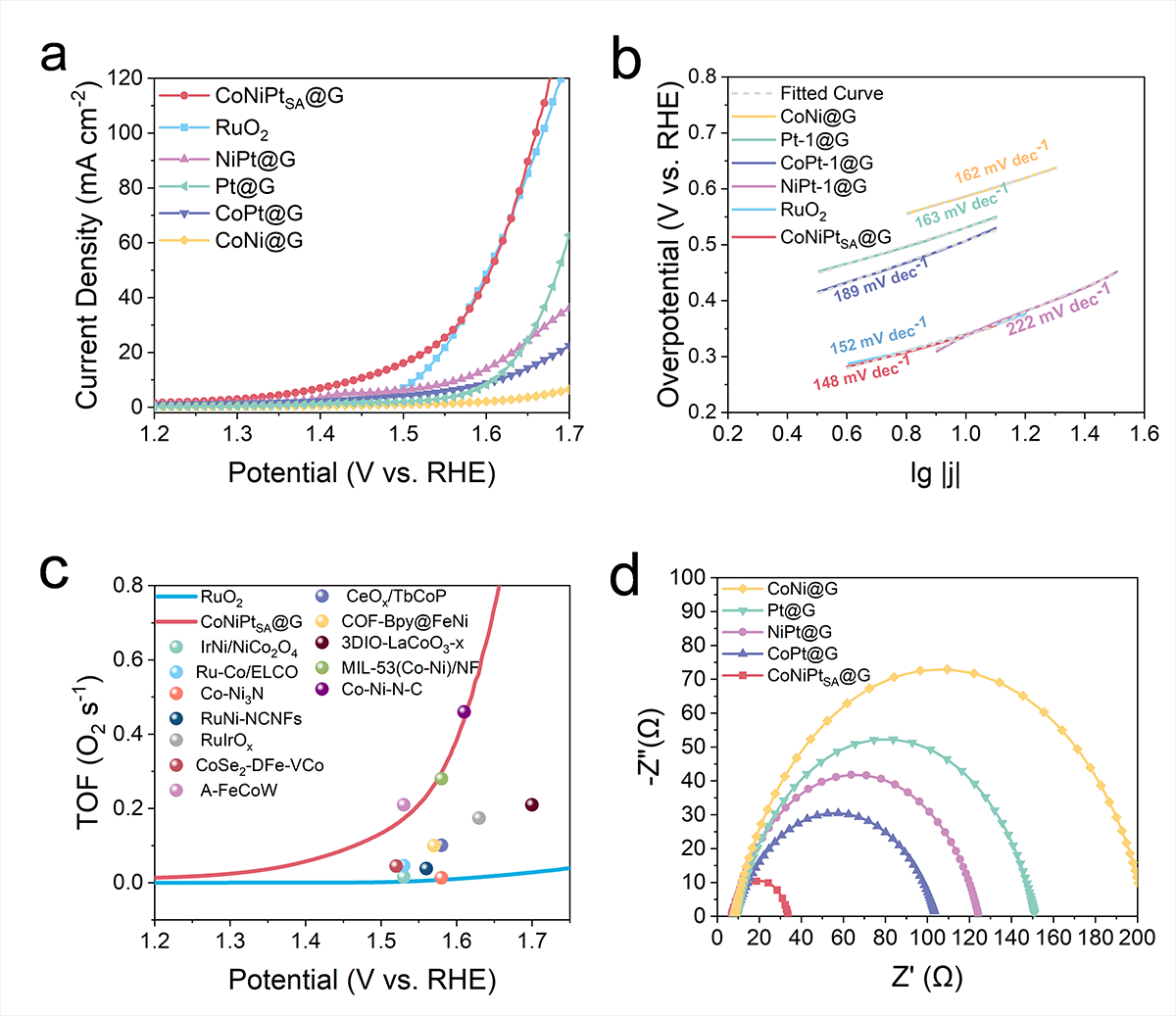
**

**Figure S17 OER Activity Evaluation. a**, Polarization curves of CoNiPt_SA_@G and other reference catalysts and RuO_2_ catalyst in the 1 M KOH electrolyte after 90% i-R compensation. **b**, Tafel slopes of CoNiPt_SA_@G and other reference catalysts. **c**, TOF values for CoNiPt_SA_@G, RuO_2_ and other reported electrocatalysts over a wide range of overpotentials. **d**, Nyquist plots at an overpotential of 0.34 V versus RHE.


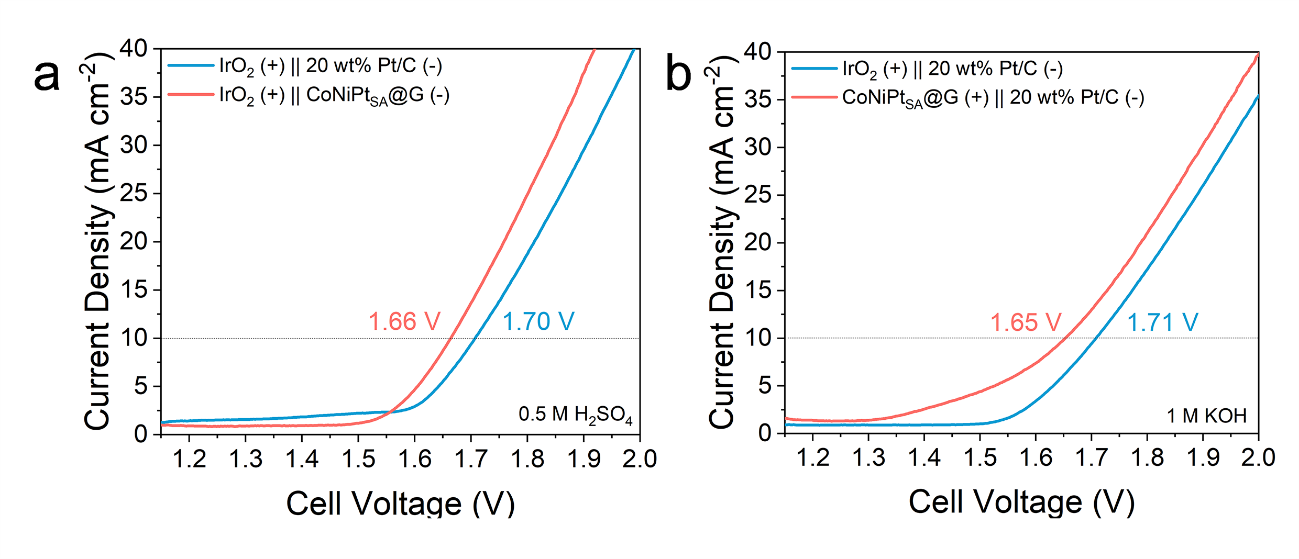


**Figure S18 Overall Water Splitting Performance Evaluation. a** Polarization curves of IrO_2_ (+) || CoNiPt_SA_@G (-) and IrO_2_ (+) || 20 wt% Pt/C (-) in 0.5 M H_2_SO_4_. **b**, Polarization curves of CoNiPt_SA_@G (+) || 20 wt% Pt/C (-)and IrO_2_ (+) || 20 wt% Pt/C (-) in 1 M KOH.

**
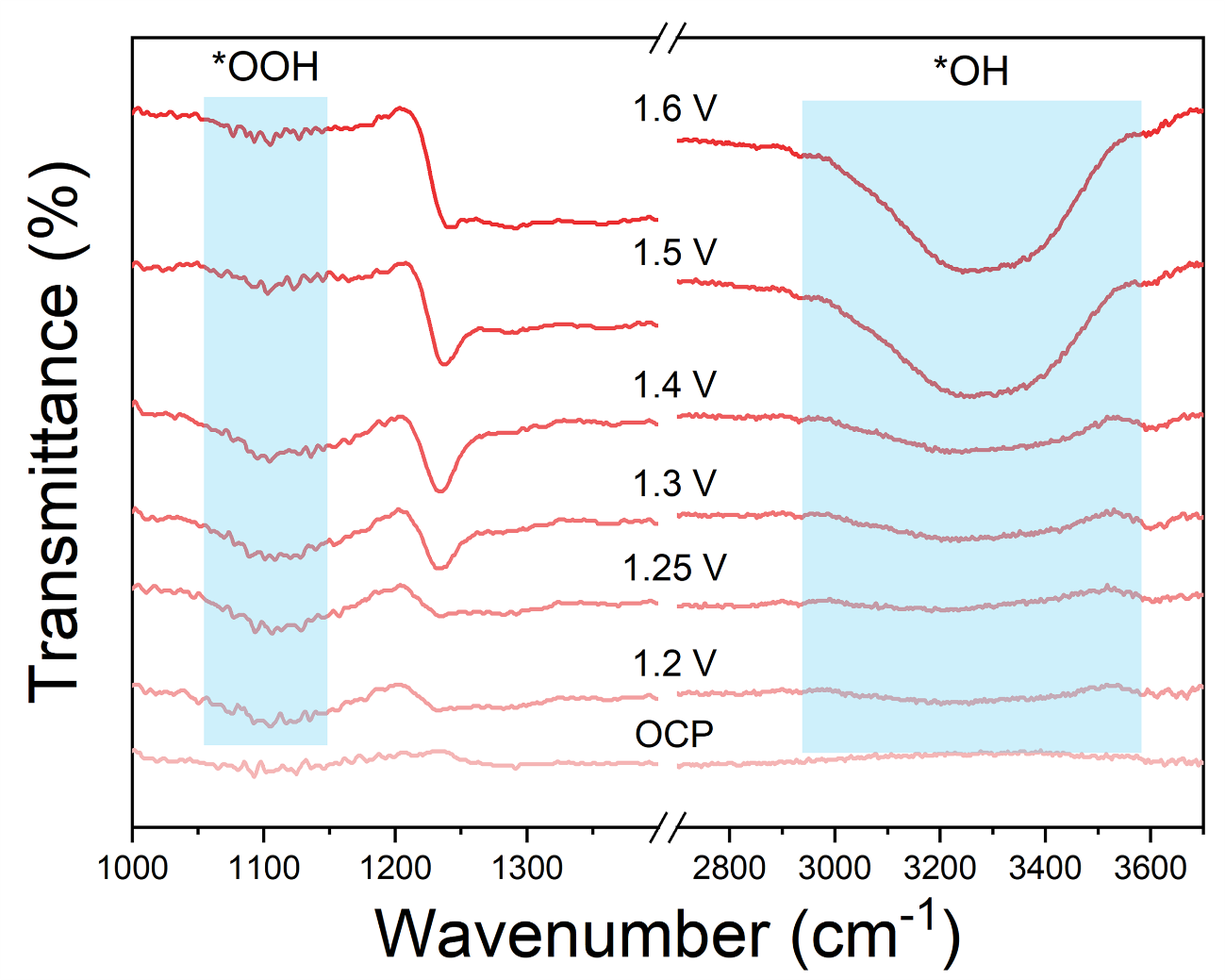
**

**Figure S19** In situ ATR-FTIR spectra of CoNiPt_SA_@G from OCP to 1.6 V versus RHE in 1 M KOH electrolyte.

**
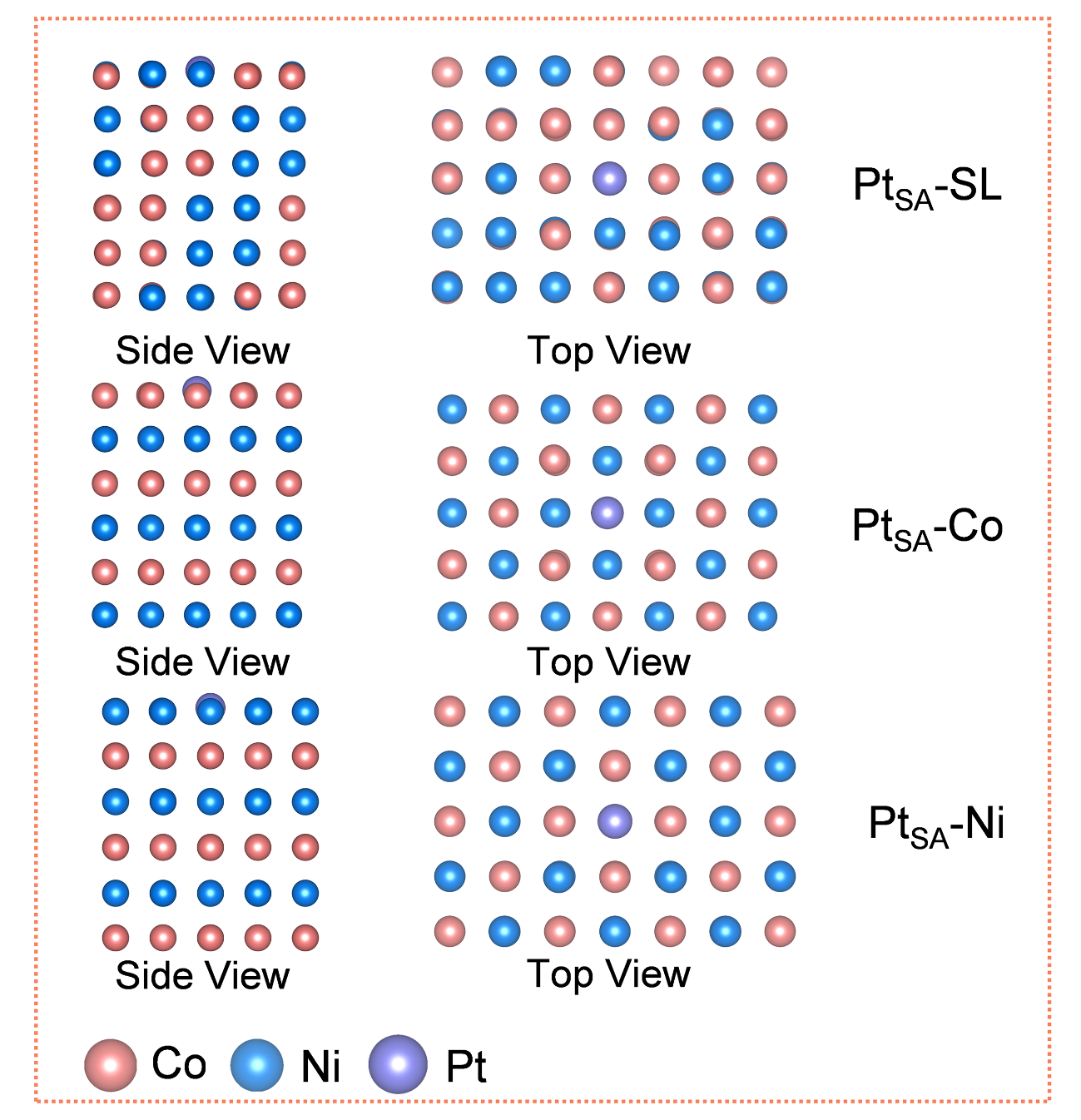
**

**Figure S20** Optimized model structure of Pt_SA_-SL, Pt_SA_-Co and Pt_SA_-Ni configurations.

**
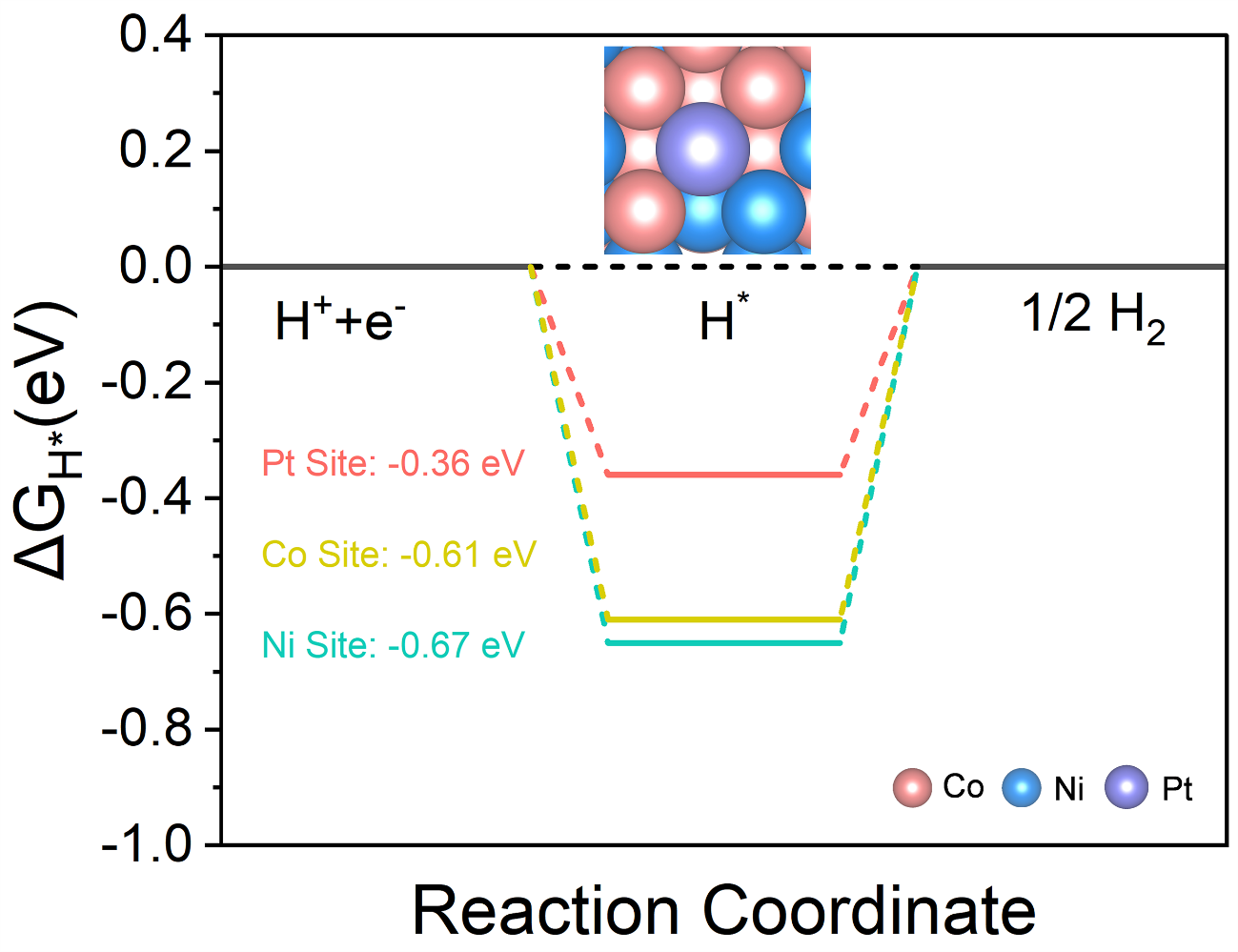
**

**Figure S21** Gibbs free energy diagrams of the H adsorption (ΔG_H*_) on the Pt, Co and Ni sites for Pt_SA_-SL configuration.

**
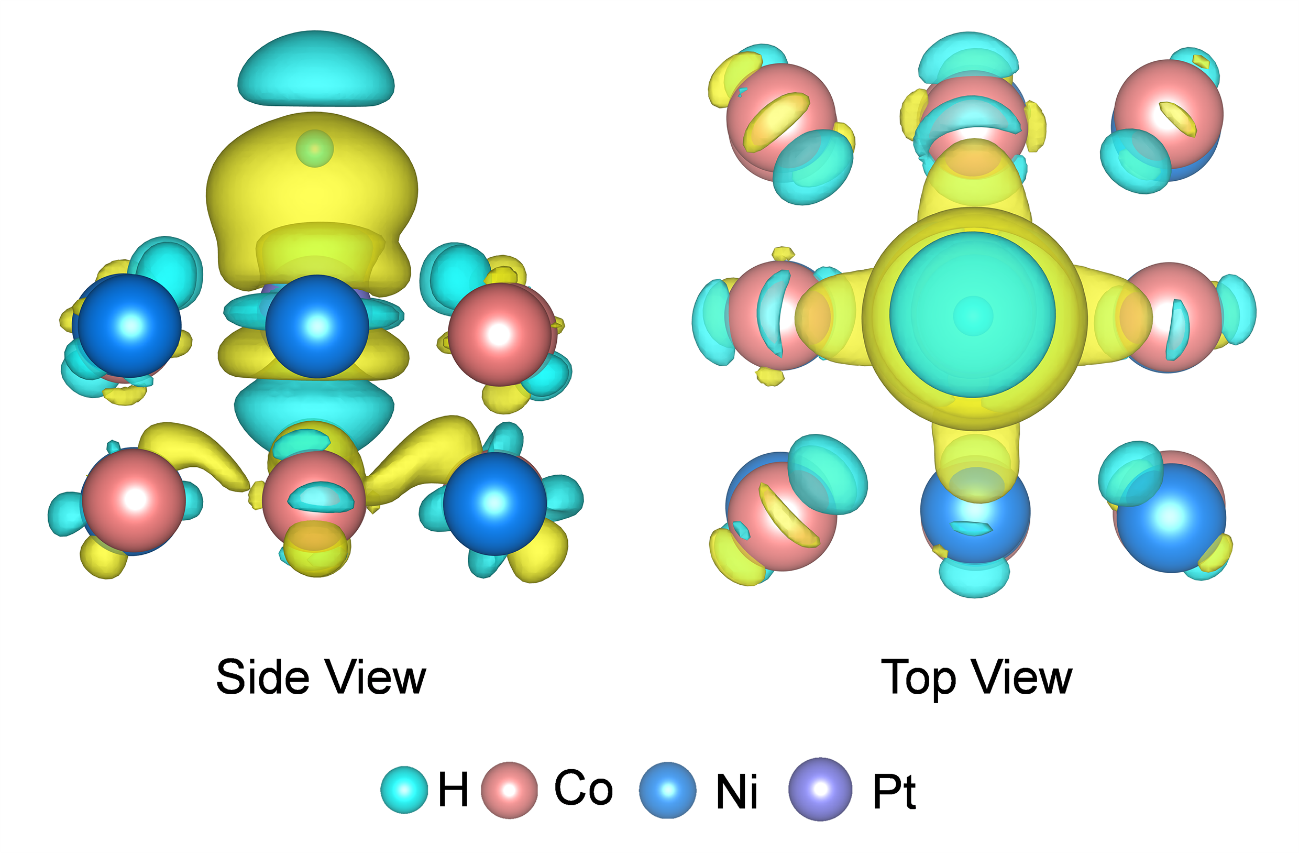
**

**Figure S22** Charge differential density map of H adsorbed Pt_SA_-SL configuration. The cyan part represents the depletion of electrons and the yellow part represents the accumulation of electrons. The isosurface value is 0.0016 e A^-3^.

**
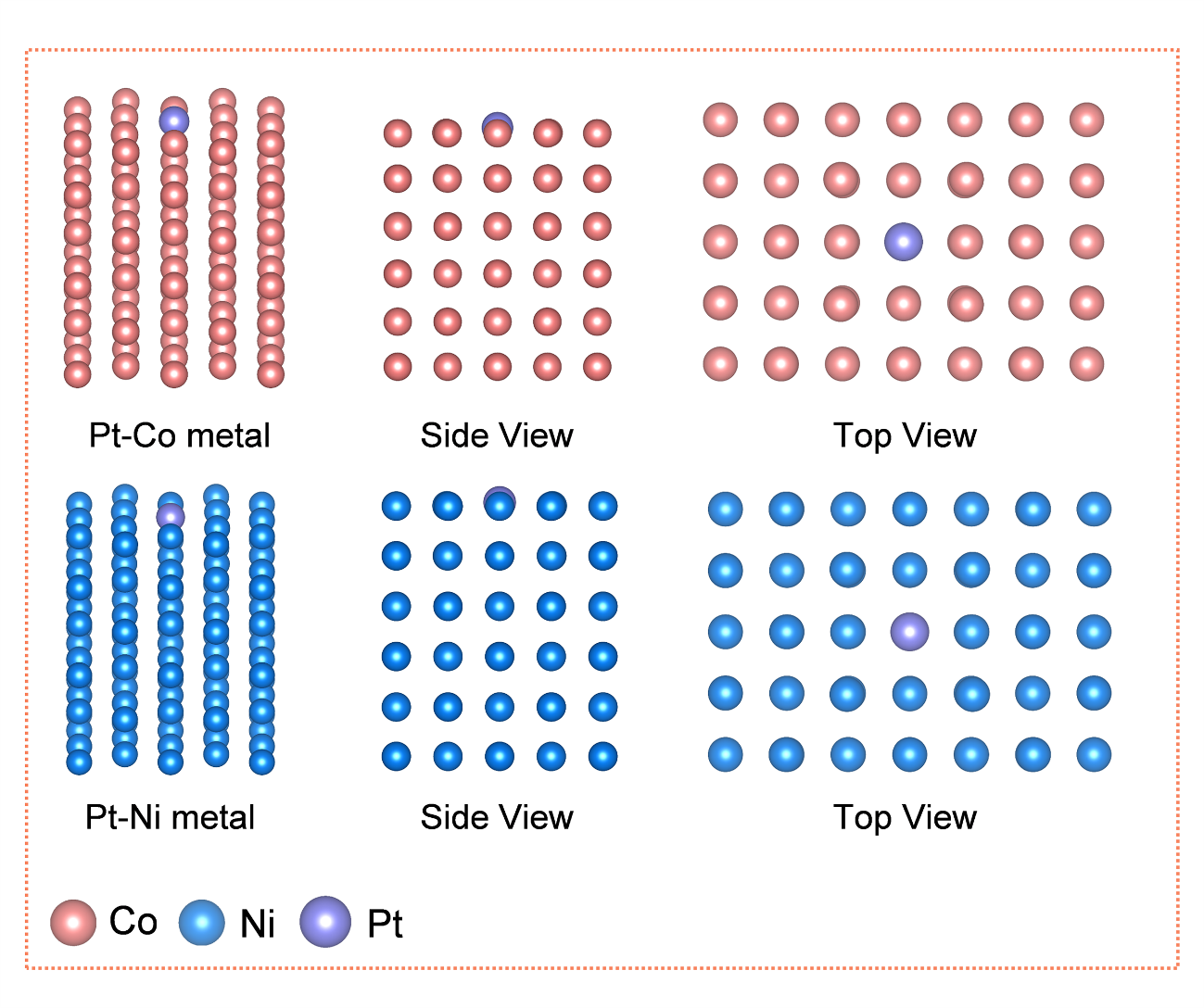
**

**Figure S23** Optimized model structure of Pt-Co metal and Pt-Ni metal configurations.

**
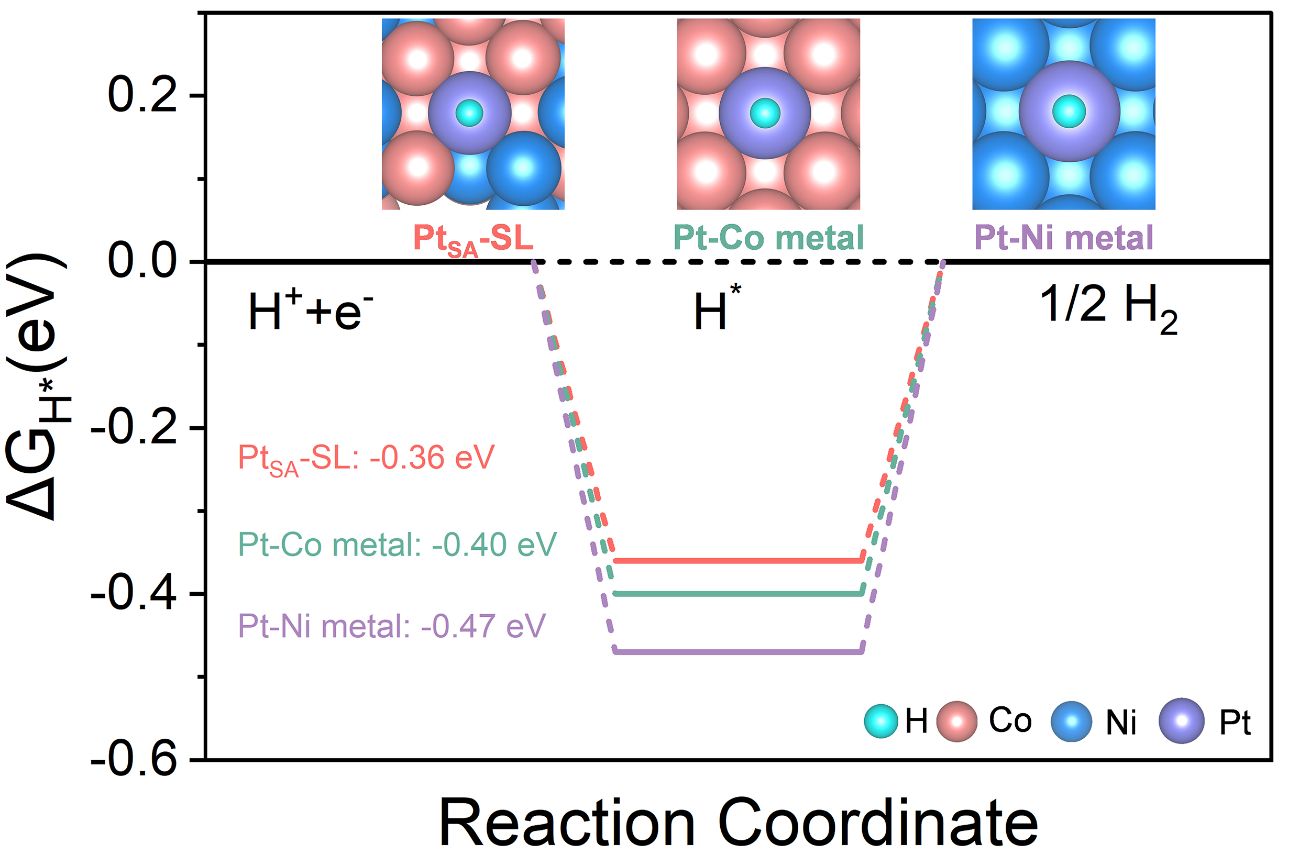
**

**Figure S24** Gibbs free energy diagrams of the H adsorption (ΔG_H*_) on the Pt site of Pt_SA_-SL, Pt-Co metal and Pt-Ni metal configurations

1. **Supplementary Tables**

**Table S1** Calculations of Pt utilization.

| **Pt Addition (g)** | **Catalyst Yield (g)** | **Pt Content (wt%)** | **Pt Utilization (%)** |
| --- | --- | --- | --- |
| 0.195 g | 3.68 | 3.82 | 72.15 |

**Table S2** Calculation of the materials cost per unit of CoNiPt_SA_@G.

| **Items** | **Price** | **Amount** | **Cost (US$)** |
| --- | --- | --- | --- |
| PtO_2_ | US$38.64/ g | 0.227 g | 8.77 |
| Co Powder | US$1.02/ g | 0.65 g | 0.66 |
| Ni Powder | US$1.24/ g | 0.65 g | 0.81 |
| Graphite Powder | US$0.003/ g | 1.3 g | 0.004 |
| Helium gas | US$179.42 (40 L, 15 MPa) | 0.026 MPa | 0.01 |
| Electricity | US$0.13/ kWh | 34 kWh | 4.42 |
| Carbon Rod | US$2.07/ stick | 1 stick | 2.07 |
| Total Cost/ Batch: US$16.74 | | | |
| Yield: 3.68 g | | | |
| Cost per Unit: US$4 .6/ g | | | |
| 20 wt% Pt/C | Cost per Unit: US$68.7/ g | | |
| RuO_2_ | Cost per Unit: US$38.6/ g | | |

**Table S3** Summary of Raman Spectra data.

| **Sample** | **Raman Shift (cm ^-1^)** | | | **Peak Intensity** | | | |
| --- | --- | --- | --- | --- | --- | --- | --- |
|  | **D** | **G** | **G’** | **I_D_** | **I_G_** | **I_D_：I_G_** | **I_G’_** |
| CoNiPt_SA_@G | 1329.65 | 1585.15 | 2649.64 | 1356.02 | 4514.35 | 0.30 | 1089.22 |

**Table S4** Comparison of the Pt areal densities of this work and other Pt SACs reported previously in the literature.

| **Number** | **Catalyst** | **Pt Areal Density (atoms nm^-2^)** | **Reference** |
| --- | --- | --- | --- |
| [1] | Pt(0.25)/TiO_2_ | 0.1 | Nat. Commun. 2024, 15, 998 |
| [2] | S-Pt-C_3_N_4_ | 0.5 | Angew. Chem. Intl. Ed. 2020, 132, 6283 |
| [3] | Pt_SA_-MNSs | 0.6 | Angew. Chem. Intl. Ed. 2019, 58, 10198 |
| [4] | Pt_1_/Al_2_O_3_ | 0.9 | Science Advances 2020, 6, 25 |
| [5] | Pt/CeO_2_ | 1.2 | Catal. Today 2024, 425, 114298 |
| [6] | Pt_1_/Fe_2_O_3_ | 1.2 | Nat. Nanotechnology 2018, 29, 204002 |
| [7] | Pt-SA/Mo-L | 3 | Adv. Mater. 2023, 36, 2305375 |
| [8] | Pt_1_/CeO_2_ | 3.9 | Angew. Chem. Int. Ed., 2022, 61, e202212338 |
| [9] | Pt_1_/NMHCS | 4 | Adv. Mater. 2021, 33, 2008599 |
| [10] | Pt_1_/PCN | 4.1 | Nat. Nanotech. 2022, 17, 174-181 |
| [11] | a-Pt_9.1_-Ni-B MNs | 5 | Sci. Adv. 2024， 10,eado2442 |
| [12] | 41.8 wt% Pt SACs/PCN | 5.6 | Nat. Commun. 2024, 15, 5675 |
| / | CoNiPt_SA_@G | 10.6 | This Work |

**Table S5** Comparison of the Pt average areal densities of this work and other Pt SACs previously reported in the literature.

| **Pt SACs** | **Pt Loading (wt%)** | **BET Surface Area (m^2^ g^-1^)** | **Pt Average Areal Density (atoms nm^-2^)** | **References** |
| --- | --- | --- | --- | --- |
| Pt_1_/NMHC | 1.59 | 475 | 0.1 | Adv. Mater. 2021, 33, 2008599 |
| Pt SA-PNPM | 2.32 | 121 | 0.6 | ACS Nano 2022, 16, 3, 4116–4125 |
| Pt-SA/pCNFs | 2.33 | 481 | 0.2 | Appl. Catal., B 2023, 15, 122898 |
| Pt_3_/WC/H | 2.80 | 432 | 0.2 | J. Colloid Interface Sci. 2025, 692, 137512 |
| mCN-Pt_1_ | 1.04 | 616 | 0.05 | Green Chem.,2025,27,8569–8575 |
| NiO–Pt_SA_ | 0.73 | 37 | 0.6 | Chem. Sci., 2024, 15, 10172 |
| Pt_SA-1.74_ /HPCNR | 1.74 | 1268 | 0.04 | Adv. Mater. 2024, 36, 2308989 |
| Pt_1_/PN-CeO_2_ | 0.36 | 109 | 0.1 | Nat. Commun. 2022, 13, 5527. |
| Pt SACs/PCN | 41.80 | 198 | 6.5 | Nat. Commun. 2024, 15, 5675 |
| CoNiPt_SA_@G | 3.82 | 76 | 1.5 | This Work |

**Table S6** Summary of XPS Pt 4f Spectra data.

| **Sample** | **Pt^0^ 4f7/2 (eV)** | **Pt^0^ 4f5/2 (eV)** | **Pt^2+^ 4f7/2 (eV)** | **Pt^2+^ 4f5/2 (eV)** |
| --- | --- | --- | --- | --- |
| CoNiPt_SA_@G | 71.1 | 74.45 | / | / |
| CoPt@G | 71.1 | 74.45 | / | / |
| NiPt@G | 70.93 | 74.28 | / | / |
| Pt@G | 70.83 | 74.18 | 72.33 | 75.68 |

**Table S7** Summary of HER performance.

| **Sample** | **η_10_(mV)** | **η_100_(mV)** | **j_0_(mA cm^-2^)** | **C_dl_ (mF cm^-2^)** | **cm_ECSA_^2^** | **R_ct_ (Ω)** |
| --- | --- | --- | --- | --- | --- | --- |
| CoNi@G | 184.37 | / | 0.06 | 5.81 | 166.00 | 1001.65 |
| Pt@G | 252.36 | 469.46 | 0.20 | 11.78 | 336.57 | 262.07 |
| NiPt@G | 107.67 | 241.61 | 0.22 | 22.19 | 634.00 | 34.65 |
| CoPt@G | 294.87 | 509.5 | 0.23 | 19.41 | 554.57 | 805.93 |
| CoNiPt_SA_@G | 22.96 | 140.54 | 0.61 | 18.64 | 532.57 | 8.08 |
| 20 wt% Pt/C | 39.66 | 141.57 | 0.53 | 20.84 | 595.42 | / |

**Table S8** Comparison of TOF values of CoNiPt_SA_@G with 20 wt% Pt/C and

other reported catalysts.

| **Sample** | **Overpotential (mV)** | **TOF (H_2_ s^-1^)** | **Reference** |
| --- | --- | --- | --- |
| RhPd-H NPs | 60 | 0.33 | ACS Nano 13, 12987-12995 (2019) |
| Ru_NP_@RuN_x_-OFC/NC | 100 | 0.49 | Appl. Catal. B: Environ. 307, 121193 (2022) |
| E-Co SAs | 100 | 0.48 | Adv. Funct. Mater. 31, 2100 (2021) |
| Pt/LiCoO_2_ | 200 | 2.25 | Angew. Chem. Int. Ed. 59, 14533–14540 (2020) |
| Ru SAs-Ni_2_P | 190 | 3 | Nano Energy 80, 105467 (2021). |
| α-Mo_2_C | 250 | 2.50 | J. Mater. Chem. A3, 8361- 8368 (2015) |
| Pt-PdO | 100 | 1.42 | ACS Sustain. Chem. & Eng. 10, 3704-3715 (2022) |
| Co-SAS-HOPNC | 200 | 3.80 | Proc. Natl. Acad. Sci. 115, 12692 (2018) |
| Mo_1_N_1_C_2_ | 150 | 1.46 | Angew. Chem. Int. Ed. 56, 16086 (2017) |
| Ni-doped graphene | 300 | 0.80 | Angew. Chem. Int. Ed. 54, 14031 (2015) |
| Pt-SA_0.056_/Mo-L | 140 | 1.20 | Adv. Mater. 2023, 2305375 |
| Pt_1_-PTh | 100 | 0.28 | Adv. Funct. Mater. 2024, 34, 2404707 |
| Pt@CoS | 100 | 0.13 | Appl. Catal. B: Environ. 2022, 315, 121534 |
| Pt SAs/Mo-PtNi/C | 200 | 0.06 | Fuel 2025, 381, 133356 |
| Pt_1_/Ni(OH)_2_ | 300 | 2.83 | Adv. Mater. 2025, 2414138 |

**Table S9** Comparison of the HER catalytic performance of CoNiPt_SA_@G with other reported Pt-based catalysts in 0.5 M H_2_SO_4_.

| **Electrocatalysts** | **η_10_ (mV)** | **Tafel Slope (mV dec^-1^)** | **References** |
| --- | --- | --- | --- |
| CoNiPt_SA_@G | 23 | 55 | This Work |
| 20 wt% Pt/C | 39 | 52 | This Work |
| CoNiIr_SA_@G | 51 | 38 | This Work |
| CNF@PtIrNiCoCe | 193 | 92 | Adv. Mater. 2023, 35, 2305222 |
| Pt_1_/NMHCS | 41 | 56 | Adv. Mater. 2021, 33, 2008599 |
| 5%Ru-MoS_2_/CNT | 50 | 62 | Adv. Sci.2019, 6, 1900090 |
| Pt-SA_0.056_/Mo-L | 31 | 31 | Adv. Mater. 2023, 36, 2305375 |
| PtS_2_/TiC | 55 | 60 | Appl. Catal. B: Environ. 2021, 293, 120227 |
| Pt@DG | 30 | 53 | J. Am. Chem. Soc. 2022, 144, 2171-2178 |
| Pt-MoS_2_ | 67 | 76 | Nano Energy 2022, 94, 106913 |
| Pt_SA_/Co_AC_O@ACTP | 61 | 43 | Adv. Mater. 2022, 34, 2206960 |
| Ag@Pt | 49 | 64 | Angew. Chem. Int. Ed. 2023, e202301065 |
| Pt/def-WO3@CFC | 42 | 61 | J. Mater. Chem. A, 2019,7, 6285-6293 |
| Ti_3_C_2_T_x_-Pt_SA_ | 38 | 45 | Nano Lett. 2022, 22, 3, 1398–1405 |
| Ir_1_@Co/NC | 60 | 119 | Angew. Chem. Int. Ed. 2019, 58, 11868 |
| Ru_SA_-N-S-Ti_3_C_2_Tx | 76 | 90 | Adv. Mater. 2019, 31, 1903841 |
| Pt@PCM | 105 | 65 | Sci. Adv. 2018, 4, eaao6657 |
| Ti_3_C_2_T_x_@Pt | 62 | 78 | Adv. Funct. Mater. 2020, 30, 2000693 |
| Pt-CoFe@NCNT/CF | 42 | 55 | Carbon 2023, 201,1068 |
| Ru-MoO_3_−x/Mo_2_AlB_2_ | 38 | 57 | Angew. Chem. Int. Ed.. 2025, e202504084 |
| Pt_1_/WCx@CNTs | 45 | 38 | Nano Lett. 2025, 25, 3066−3074 |
| Pt_1_/NBP | 143 | 69 | Chin. J. Catal. 2025, 69, 259-270 |
| Pt SAs/Mo-PtNi/C | 38 | 44 | Fuel 2025, 381, 133356 |
| 1Pt/VS_2_/CP | 77 | 40 | ACS NANO 2020, 14, 5600-5608 |
| Pt-CNTs | 41 | 49 | J. Energy Chem. 2020, 51, 280-284 |
| Pt SA/m-WO_3_−x | 38 | 45 | Angew. Chem. Int. Ed. 2019, 58, 16038. |
| Pt-GDY2 | 66 | 46 | Angew. Chem., Int. Ed. 2018, 57, 9382-9386. |
| Ti_3_C2T_x_-N-Pt_SA_ | 86 | 61 | Nano Lett. 2022, 22, 3, 1398–1405 |

**Table S10** Element content in the electrolyte after stability test.

| **Element** | **Content（μg/L)** |
| --- | --- |
| Co | 326.26 |
| Ni | 311.35 |
| Pt | 0.67 |

**Table S11** Summary of OER performance.

| **Sample** | **η_10_(mV)** | **R_ct_ (Ω)** |
| --- | --- | --- |
| CoNi@G | 503 | 195 |
| Pt@G | 379 | 141 |
| NiPt@G | 337 | 117 |
| CoPt@G | 283 | 95 |
| CoNiPt_SA_@G | 213 | 27 |
| RuO_2_ | 273 | / |

**Table S12** Comparison of OER TOF values of CoNiPt_SA_@G and

other reported catalysts.

| **Sample** | **Potential (V)** | **TOF (O_2_ s^-1^)** | **Reference** |
| --- | --- | --- | --- |
| IrNi/NiCo_2_O_4_ | 1.53 | 0.016 | Sci. China Mater. 2017, 60, 119 |
| Ru-Co/ELCO | 1.53 | 0.046 | Angew. Chem. 2022, 134, e202205946 |
| Co-Ni_3_N | 1.58 | 0.0134 | Adv. Mater. 2018, 30, 1705516 |
| RuNi-NCNFs | 1.56 | 0.038 | Adv. Sci. 2020, 7, 1901833 |
| RuIrOx | 1.63 | 0.174 | Adv. Mater. 2020, 32, 2002297 |
| CoSe_2_-DFe-VCo | 1.53 | 0.045 | Nat. Commun. 2020, 11, 1664 |
| A-FeCoW | 1.53 | 0.21 | Science 2016, 352, 333-337 |
| CeO_x_/TbCoP | 1.58 | 0.101 | J. Alloy. Compd. 2024, 972, 172820 |
| COF-Bpy@FeNi | 1.57 | 0.1 | Rare Met. 2024, 43, 3096–3106 |
| 3DIO-LaCoO_3_-x | 1.70 | 0.21 | J. Power Sources 2020, 478, 228748 |
| MIL-53(Co-Ni)/NF | 1.58 | 0.28 | Colloids Surf. A Physicochem. Eng. Asp. 2022, 647, 129041 |
| Co-Ni-N-C | 1.61 | 0.46 | Nat Energy 2021, 6, 1054–1066 |
| CoNiPt_SA_@G | 1.53 | 0.17 | This work |
| RuO_2_ | 1.53 | 0.00164 | This work |

**References:**

[1] B. Ravel, M. Newville, *Journal of Synchrotron Radiation* **2005**, *12*, 537.

[2] J. Timoshenko, A. Kuzmin, *COMPUTER PHYSICS COMMUNICATIONS* **2009**, *180*, 920.

[3] S. Liu, Z. Hu, Y. Wu, J. Zhang, Y. Zhang, B. Cui, C. Liu, S. Hu, N. Zhao, X. Han, A. Cao, Y. Chen, Y. Deng, W. Hu, *Advanced Materials* **2020**, *32*, 2006034.

[4] Q. Wang, X. Huang, Z. L. Zhao, M. Wang, B. Xiang, J. Li, Z. Feng, H. Xu, M. Gu, *Journal of the American Chemical Society* **2020**, *142*, 7425.

[5] C. C. L. McCrory, S. Jung, I. M. Ferrer, S. M. Chatman, J. C. Peters, T. F. Jaramillo, *Journal of the American Chemical Society* **2015**, *137*, 4347.

[6] L. Zhuang, Y. Jia, H. Liu, X. Wang, R. K. Hocking, H. Liu, J. Chen, L. Ge, L. Zhang, M. Li, C.-L. Dong, Y.-C. Huang, S. Shen, D. Yang, Z. Zhu, X. Yao, *Advanced Materials* **2019**, *31*, 1805581.

[7] G. Kresse, D. Joubert, *Phys Rev B* **1999**, *59*, 1758.

[8] S. Grimme, *J Comput Chem* **2006**, *27*, 1787.

[9] J. Zhang, Y. Zhao, X. Guo, C. Chen, C.-L. Dong, R.-S. Liu, C.-P. Han, Y. Li, Y. Gogotsi, G. Wang, *Nat Catal* **2018**, *1*, 985.

[10] L. D. Rafailović, C. Gammer, C. Rentenberger, C. Kleber, A. H. Whitehead, B. Gollas, H.-P. Karnthaler, *Physical Chemistry Chemical Physics* **2012**, *14*, 972.

[11] Y. Wang, P. Ren, J. Hu, Y. Tu, Z. Gong, Y. Cui, Y. Zheng, M. Chen, W. Zhang, C. Ma, L. Yu, F. Yang, Y. Wang, X. Bao, D. Deng, *Nat Commun* **2021**, *12*, 5814.

[12] J. Feng, F. Lv, W. Zhang, P. Li, K. Wang, C. Yang, B. Wang, Y. Yang, J. Zhou, F. Lin, G.-C. Wang, S. Guo, *Advanced Materials* **2017**, *29*, 1703798.

[13] X. Liu, Y. Zhou, J. Lin, X. Xiao, Z. Wang, L. Jia, M. Li, K. Yang, J. Fan, weiwei Yang, G. Li, *Angewandte Chemie International Edition* **2024**, *n/a*, e202406650.

[14] Y. Zhu, T. Cao, C. Cao, J. Luo, W. Chen, L. Zheng, J. Dong, J. Zhang, Y. Han, Z. Li, C. Chen, Q. Peng, D. Wang, Y. Li, *ACS Catalysis* **2018**, *8*, 10004.
